# Observing non-ergodicity due to kinetic constraints in tilted Fermi-Hubbard chains

**DOI:** 10.1038/s41467-021-24726-0

**Published:** 2021-07-23

**Authors:** Sebastian Scherg, Thomas Kohlert, Pablo Sala, Frank Pollmann, Bharath Hebbe Madhusudhana, Immanuel Bloch, Monika Aidelsburger

**Affiliations:** 1 Fakultät für Physik, Ludwig-Maximilians-Universität München, Munich, Germany; 2 Max-Planck-Institut für Quantenoptik, Garching, Germany; 3 Munich Center for Quantum Science and Technology (MCQST), München, Germany; 4 Department of Physics, Technical University of Munich, Garching, Germany

**Keywords:** Ultracold gases, Electronic properties and materials, Quantum simulation, Statistical physics

## Abstract

The thermalization of isolated quantum many-body systems is deeply related to fundamental questions of quantum information theory. While integrable or many-body localized systems display non-ergodic behavior due to extensively many conserved quantities, recent theoretical studies have identified a rich variety of more exotic phenomena in between these two extreme limits. The tilted one-dimensional Fermi-Hubbard model, which is readily accessible in experiments with ultracold atoms, emerged as an intriguing playground to study non-ergodic behavior in a clean disorder-free system. While non-ergodic behavior was established theoretically in certain limiting cases, there is no complete understanding of the complex thermalization properties of this model. In this work, we experimentally study the relaxation of an initial charge-density wave and find a remarkably long-lived initial-state memory over a wide range of parameters. Our observations are well reproduced by numerical simulations of a clean system. Using analytical calculations we further provide a detailed microscopic understanding of this behavior, which can be attributed to emergent kinetic constraints.

## Abstract (web summary)

It was predicted that complex thermalizing behaviour can arise in many-body systems in the absence of disorder. Here, the authors observe non-ergodic dynamics in a tilted optical lattice that is distinct from previously studied regimes, and propose a microscopic mechanism that is due to emergent kinetic constrains.

## Introduction

Understanding the complex out-of-equilibrium dynamics of quantum many-body systems is central to a number of research areas ranging from statistical physics to quantum information theory^1–3^. State-of-the-art experimental platforms are now able to test novel theoretical concepts and approximate descriptions based on experimental observations. Important experimental results were obtained in particular with integrable^4^ or many-body localized (MBL)^5–7^ systems. Both phenomena emerge due to the existence of extensively many conserved quantities and have been of considerable interest, because they break the eigenstate thermalization hypothesis, which assumes that each individual eigenstate behaves locally like a thermal ensemble and is believed to hold for generic ergodic systems^8–10^.

In between the two extreme limits of ergodic and localizing dynamics there exists a rich variety of more complex thermalizing behavior. Models with many-body scar states, e.g., host a vanishing fraction of non-thermal eigenstates embedded within an otherwise thermal spectrum^11–16^. They exhibit a weak form of ergodicity-breaking, that strongly depends on the initial state, as has been observed with Rydberg atoms^14,17,18^. More recently, a whole new class of models has been suggested, where the presence of only few conserved quantities, in particular dipole conservation, results in non-ergodic dynamics due to an emergent fragmentation of the Hilbert space into exponentially many disconnected subspaces^19–22^. Fragmented models offer an alternative view on a central open question, namely if many-body localization can occur in translationally-invariant models without disorder^23–29^.

**Fig. 1:**
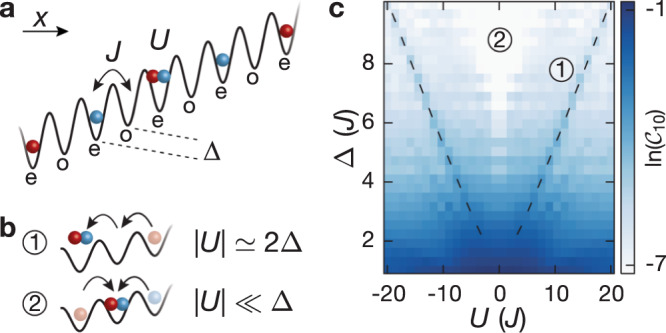
Illustration of the experimental setup and the structure of the Hilbert space. **a** Schematic of the tilted 1D Fermi-Hubbard model (with odd *o* and even *e* sites) with tunneling *J*, on-site interaction *U* and spin-dependent tilt Δ_↑_, Δ_↓_ (spin-up red, spin-down blue). **b** Dominant resonant tunneling processes for different regimes. **c** Finite-time connectivity ${{{{\mathcal{C}}}}}_{\epsilon }$ (for a cut-off *ϵ* = 10%) defined as the fraction of states that participate in the dynamics up to an evolution time ${T}_{{{{\mathcal{N}}}}}=1000\tau$ (main text, “Methods”). The calculation was performed for a Néel-ordered singlon CDW initial state, using exact diagonalization (ED) with system size *L* = 13 and Δ_↑_ = Δ_↓_ ≡ Δ. In the large-tilt limit, Δ/*J* → *∞*, we find emergent strongly-fragmented effective Hamiltonians for regime ① and ② (see Supplementary Note 3 and 4).

In this work, we study non-ergodic behavior in the disorder-free tilted one-dimensional (1D) Fermi-Hubbard model (Fig. 1a), which lies at the interface of MBL and Hilbert-space fragmentation. In the presence of additional weak disorder or harmonic confinement, theoretical studies have found characteristic MBL phenomenology, known as Stark MBL^30–34^. This, however, does not hold for a clean system with pure linear potential^30,33^. While conventional MBL predicts localization for any typical initial state, we do not expect this to hold for our system, where resonances can occur between interaction and tilt energies (regime ① in Fig. 1b). Intriguingly, it has been predicted, that in the limit of large tilts, Δ ≫ *J*, ∣*U*∣, non-ergodicity may still occur despite the absence of disorder. In this regime, the large tilt energy imposes kinetic constraints, which result in an emergent dipole conservation^19,20,22,31,33^. This emergent behavior is in fact governed by a fragmented Hamiltonian resulting in non-ergodic dynamics. Starting from an initial charge-density wave (CDW) of singlons (singly-occupied site), we study relaxation dynamics in the tilted 1D Fermi-Hubbard model for a large range of interaction strengths and moderate values of the tilt (Δ < 4*J*), where none of the two mechanisms described above should apply and where naively one may expect the system to thermalize^35,36^. At short times we observe coherent dynamics due to Bloch oscillations, whose amplitude strongly depends on the Hubbard interactions. Surprisingly we find that after intermediate times and even close to resonance (regime ①), the evolution converges to a steady-state, that persists for long evolution times up to 700 tunneling times, signaling a robust memory of the initial CDW throughout.

Using numerical calculations we show that the observed non-ergodicity cannot be explained by the phenomenon of Stark-MBL, i.e., the robust memory is not due to experimental imperfections, such as residual harmonic confinement or disorder, and the bipartite entanglement entropy does not exhibit the characteristic behavior of MBL systems^30,37^ (Supplementary Fig. 5). Hence, non-ergodicity appears to have a different origin, despite similar experimental signatures. This raises the question about the origin of the observed non-ergodicity. We construct effective Hamiltonians in two distinct regimes (① and ②, Fig. 1b) by taking the large tilt limit and find strongly-fragmented Hamiltonians in both cases (Supplementary Note 3). While these models are only expected to describe the dynamics at large tilt values and for intermediate times (on the order of a few tens of tunneling times), they allow us to identify the microscopic processes that initiate dynamics at short times (Fig. 1b). In both regimes these are correlated tunneling processes, which result in the formation of doublons (doubly-occupied sites), either resonantly (regime ①) or detuned by the Hubbard interaction energy *U* (regime ②). Higher-order terms are expected to eventually drive the system towards thermalization^19^. However, we are able to show that energy penalties for the second- or higher-order tunneling processes, which occur naturally in the model, render these dynamics inefficient. This results in extremely slow relaxation (Supplementary Note 3), which appears stable for > 10^4^ tunneling times in our exact diagonalization studies of small systems, in agreement with our experimental observations (Supplementary Fig. 4).

In order to characterize the dynamics across the whole parameter regime studied experimentally, we compute the finite-time connectivity of our initial CDW state ${{{{\mathcal{C}}}}}_{\epsilon }=\dim ({{{{\mathcal{N}}}}}_{\epsilon })/\dim ({{{\mathcal{H}}}})$, which is defined by the fraction of states that participate in the time evolution up to a finite time ${T}_{{{{\mathcal{N}}}}}$; here ${{{{\mathcal{N}}}}}_{\epsilon }$ denotes the subspace in the complete Hilbert-space ${{{\mathcal{H}}}}$, which is defined, such that the residual overlap of the time-evolved state $\left|\psi (t)\right\rangle$ outside of ${{{{\mathcal{N}}}}}_{\epsilon }$ is at most *ϵ* at any time $t\le {T}_{{{{\mathcal{N}}}}}$ (Methods). The value of *ϵ* is typically chosen between 1 and 10%. The finite-time connectivity can be understood as a measure of non-ergodicity, similar to the more conventional return probability or other multifractality measures^38^. While effective Hamiltonians can only be derived explicitly in certain limits, the numerical construction is applicable in the whole parameter regime probed in this work (Fig. 1c). We find that the finite-time connectivity vanishes in the thermodynamic limit for all parameters, suggesting that only a small fraction of the states participates in the dynamics, signaling non-ergodic behavior. Our results suggest that the emergent kinetic constraints result in transient non-ergodic behavior across the whole parameter range studied in this work. We further show analytically that the relevant microscopic constraints in the resonant ① regime give rise to Hilbert-space fragmentation in the large tilt limit (Supplementary Note 4).

## Results

The experimental setup consists of a degenerate Fermi gas of 50(5) × 10^3^ ^40^K atoms that is prepared in an equal mixture of two spin components $\left|\uparrow \right\rangle =\left|{m}_{F}=-7/2\right\rangle$ and $\left|\downarrow \right\rangle =\left|{m}_{F}=-9/2\right\rangle$ in the *F* = 9/2 ground-state hyperfine manifold. The atoms are loaded into a 3D optical lattice with lattice constant *d*_*s*_ = 266 nm along the *x* direction and deep transverse lattices, with constant *d*_⊥_ = 369 nm, to isolate the 1D chains along *x* (“Methods”). The central 1D chains have a length of about 290 lattice sites. The residual coupling along the transverse directions is less than 3 × 10^−4^*J*. The dynamics along *x* is described by the tilted 1D Fermi-Hubbard model

1 $$\hat{H}=	 \,\mathop{\sum}\limits_{i,\sigma =\uparrow ,\downarrow }\, \left(-J{\hat{c}}_{i,\sigma }^{{\dagger} }{\hat{c}}_{i+1,\sigma }+{{{\rm{h}}}}.{{{\rm{c}}}}.+{{{\Delta }}}_{\sigma }i{\hat{n}}_{i,\sigma }\right)\\ \,	+\;U\mathop{\sum}\limits_{i}{\hat{n}}_{i,\uparrow }{\hat{n}}_{i,\downarrow },$$

where ${\hat{c}}_{i\sigma }^{{\dagger} }$ (${\hat{c}}_{i\sigma }$) is the fermionic creation (annihilation) operator and ${\hat{n}}_{i,\sigma }={\hat{c}}_{i\sigma }^{{\dagger} }{\hat{c}}_{i\sigma }$. The on-site interaction strength *U* is controlled by a Feshbach resonance centered at 202.1 G and a magnetic field gradient is used to create the tilt Δ_*σ*_, with Δ_↑_ ≃ 0.9Δ_↓_. The weak spin-dependence arises due to the different *m*_*F*_ quantum numbers (Supplementary Note 9 and 11). The initial state for all subsequent measurements is a CDW of singlons on even sites, which is prepared using a bichromatic optical superlattice (Supplementary Note 7). The initial state can be described as an incoherent mixture of site-localized particles with random spin configuration (“Methods”). The subsequent evolution is monitored by extracting the spin-resolved imbalance ${{{{\mathcal{I}}}}}^{\sigma }=({N}_{e}^{\sigma }-{N}_{o}^{\sigma })/{N}^{\sigma }$; here ${N}_{e(o)}^{\sigma }$ denotes the total number of spin-*σ* atoms on even (odd) sites and ${N}^{\sigma }={N}_{e}^{\sigma }+{N}_{o}^{\sigma }$. A non-zero steady-state imbalance signals a memory of the initial state, where ${{{{\mathcal{I}}}}}^{\sigma }(t=0)=1$.

**Fig. 2:**
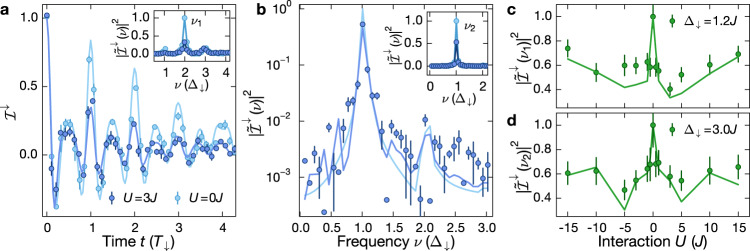
Short-time interacting Bloch oscillations. **a** Imbalance ${{{{\mathcal{I}}}}}^{\downarrow }$ for *U* = 0*J* (spin-polarized gas, light blue) and *U* = 3*J* (spin-resolved measurement, dark blue) with *J*/*h* = 0.88(2) kHz and Δ_↓_ = 1.22(1)*J*. Inset: Power spectral density (PSD) $| \tilde{{{{\mathcal{I}}}}}(\nu ){| }^{2}$ of the time traces shown in the main panel, normalized to the maximum of the non-interacting spectrum; *ν*_1_ = 2Δ_↓_/*h* indicates the dominant frequency component. **b** PSD $| \tilde{{{{\mathcal{I}}}}}(\nu ){| }^{2}$ for *U* = 3*J* (spin-resolved measurement, dark blue), normalized to the maximum of the non-interacting spectrum; *J*/*h* = 0.54(1) kHz and Δ_↓_ = 2.96(3)*J*. The data were obtained from time-traces as in (**a**). Inset: PSD as in the main panel and for *U* = 0*J* (spin-polarized gas, light blue). *ν*_2_ = Δ_↓_/*h* indicates the dominant frequency. **c**, **d** Interaction scan of the peak power spectral density $| \tilde{{{{\mathcal{I}}}}}({\nu }_{j}){| }^{2}$ evaluated by summing the PSD in a window of ±3 data points around the dominant frequency *ν*_*j*_, *j* = {1, 2} at **c** Δ_**↓**_ = 1.22(1)*J* and **d** Δ_↓_ = 2.96(3)*J* obtained from traces as in (**a**). Each data point in (**a**, **b**) consists of four independent measurements and the error bars denote the standard error of the mean (SEM). Solid lines in all panels are numerical simulations using TEBD (“Methods”).

In a first set of measurements we study the effect of interactions on the coherent short-time dynamics. In a tilted lattice an initially localized particle exhibits Bloch oscillations^39^, with a characteristic period *T*_*σ*_ = *h*/Δ_*σ*_, set by the spin-dependent tilt. In the presence of interactions, Bloch oscillations persist, showing a rich variety of dynamics, such as interaction-induced dephasing and amplitude modulation^40–45^. Here, we use the spin-resolved imbalance to probe real-space Bloch oscillations in a parity-projected manner. In the non-interacting limit the time-dependence can be computed analytically:

2 $${{{{\mathcal{I}}}}}^{\sigma }(t)={{{{\mathcal{J}}}}}_{0}\left(\frac{8J}{{{{\Delta }}}_{\sigma }}\sin \left(\frac{\pi {{{\Delta }}}_{\sigma }t}{h}\right)\right),$$

which enables a precise calibration of the model parameters Δ_*σ*_ and *J* (Fig. 2a) at short times. Here, ${{{{\mathcal{J}}}}}_{0}$ denotes the 0th-order Bessel function of the first kind. The dephasing of the oscillations is caused by a residual harmonic confinement that results in a weak local variation *δT*_*σ*_ of the Bloch oscillation period *T*_*σ*_ between adjacent sites. An upper bound for the trap frequency *ω*_*h*_/(2*π*) = 39 Hz was extracted from independent measurements (Supplementary Note 11) and corresponds to *δT*_*σ*_/*T*_*σ*_ ≪ 10^−3^. Since the imbalance dynamics for both spin components is very similar (see Supplementary Fig. 10), we focus on one component ${{{{\mathcal{I}}}}}^{\downarrow }$.

For weak tilt values, Δ_↓_ = 1.2*J*, we find that the dynamics of the interacting spin-mixture (*U* = 3*J*) exhibits the same dominant frequency components as the non-interacting Bloch oscillations, while the dephasing is strongly enhanced. This can be seen more directly by calculating the power spectral density (PSD) of the imbalance $| {\tilde{{{{\mathcal{I}}}}}}^{\sigma }(\nu ){| }^{2}$ (inset of Fig. 2a). We find three distinct peaks in the spectrum, the Bloch frequency Δ_↓_ and an admixture of two higher harmonics with the largest spectral weight in the second harmonic at *ν*_1_ = 2Δ_↓_/*h*. For *U* = 3*J* its weight is decreased by 70% compared to the non-interacting case. The higher-order harmonics originate from the real-space evolution within one Bloch cycle and are determined by the Bloch oscillation amplitude *A*_*σ*_/*d*_*s*_ = 4*J*/Δ_*σ*_. We anticipate frequency components at integer multiples of Δ_*σ*_, with an upper bound determined by *A*_*σ*_/*d*_*s*_, in agreement with our data.

Interaction effects are expected to be less relevant once the Bloch oscillation amplitude is smaller than one site, resulting in negligible overlap between neighboring particles for our CDW initial state. In Fig. 2b we show the PSD of the coherent short-time dynamics for Δ_↓_ = 3.0*J*. While the largest spectral weight of the PSD is now contained in the Bloch frequency *ν*_2_ = Δ_↓_/*h*, the reduction is still about 50% compared to the non-interacting case. Indeed, the spectral weight is a sensitive measure of the interaction-induced dephasing. Moreover, the on-site interactions lift the degeneracy of the energy levels in the Wannier-Stark spectrum, which results in additional frequency components in the PSD. For our parameters (Fig. 2b) they occur at ≈ *ν*_2_ ± 0.5Δ_↓_/*h* in the time-evolving block decimation (TEBD) simulations^46–48^, which is consistent with our data.

The sensitivity of the coherent short-time dynamics on the interaction strength is further highlighted by the strong interaction-dependence of the peak power spectral density (PPSD) $| \tilde{{{{\mathcal{I}}}}}({\nu }_{j}){| }^{2}$ of the respective dominant frequency components *ν*_*j*_, *j* = {1, 2} (Fig. 2c, d). We find a sharp decrease of the PPSD by about 40% already for small interaction strength *U* =± 0.5*J* for Δ_*σ*_ = 1.2*J*. After reaching a global minimum at intermediate interaction strength, it slowly recovers to the non-interacting value in the limit of large interactions.

**Fig. 3:**
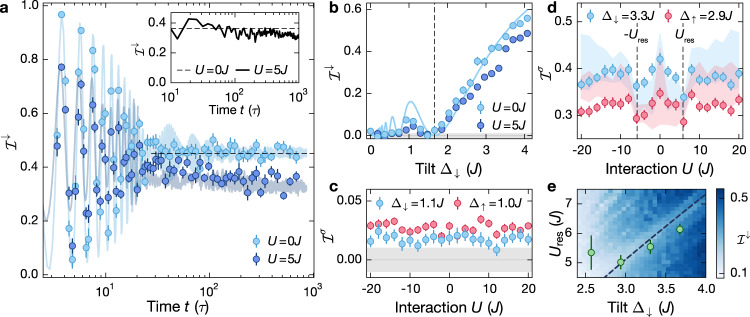
Long-time dynamics. **a** Imbalance time traces at Δ_↓_ = 3.30(3)*J* and *J*/*h* = 0.54(1)kHz for *U* = 0*J* (spin-polarized, light blue) and *U* = 5*J* (spin-resolved measurement, dark blue). The shaded trace is an ED calculation for *L* = 16 (“Methods”). Each data point is averaged over 12 individual experimental realizations. Inset: ED calculation for *L* = 16 in a clean system with Δ_↓_ = Δ_↑_ = 3*J*, *ω*_*h*_ = 0 and *U* = 5*J* using a Néel-ordered initial CDW. The dashed lines show the analytic prediction for the non-interacting steady-state imbalance [Eq. (3)]. **b** Steady-state imbalance versus Δ_↓_ measured at *U* = 0*J* (spin-polarized, light blue) and *U* = 5*J* (spin-resolved measurement, dark blue). Each data point is averaged over ten equally spaced times in a time window between 70*τ* and 100*τ* (*U* = 0*J*) and 340*τ* and 370*τ* (*U* = 5*J*). The solid line shows the analytic prediction for ${{{{\mathcal{I}}}}}^{\downarrow }$ [Eq. (3)] and the dashed line indicates the first root of the Bessel function at Δ_↓_ ≈ 1.5*J*. **c** Spin-resolved steady-state imbalance versus interaction strength at Δ_↓_ = 1.10(1)*J*. Each point is averaged over ten time steps equally spaced between 170*τ* and 200*τ*. **d** Spin-resolved steady-state imbalance versus interaction strength as in (**c**) for Δ_↓_ = 3.30(3)*J*. The shaded trace is an ED simulation, which is averaged over the same time steps as in (**c**) and where the width indicates the 1*σ* standard deviation. **e** Resonances extracted from interaction scans for *U* > 0 as in (**d**) for different tilt values (Supplementary Note 12). The color plot shows ED calculations for the same parameters as in the experiment, but with *ω*_*h*_ = 0, for *L* = 13 sites. The dashed line indicates the analytic prediction for the resonance ${U}_{{{{\rm{res}}}}}\simeq 2{{{\Delta }}}_{\downarrow }-8{J}^{2}/(3{{{\Delta }}}_{\downarrow })$. The gray shaded area in (b),(c) indicates our calibrated detection resolution. In all panels error bars denote the SEM.

For long enough evolution times, the coherent Bloch oscillations are dephased and a finite steady-state imbalance develops in the non-interacting limit (Fig. 3a). Note that, if the dephasing was solely due to residual harmonic confinement, we would expect a coherent revival of the oscillations, which is suppressed in our experiment by additional dephasing mechanisms and ensemble averaging. The observed finite steady-state imbalance is caused by Wannier-Stark localization and can be computed analytically by time averaging the short-time dynamics:

3 $${{{{\mathcal{I}}}}}^{\sigma }=\mathop{{{{\rm{lim}}}}}\limits_{T\to \infty }\frac{1}{T}\int\nolimits_{0}^{T}{{{{\mathcal{I}}}}}^{\sigma }(t)\ dt={{{{\mathcal{J}}}}}_{0}^{2}\left(\frac{4J}{{{{\Delta }}}_{\sigma }}\right).$$

Excellent agreement between our data and the analytical result provides strong evidence that the effect of the harmonic confinement is negligible for the late-time steady-state imbalance, in contrast to previous fermionic transport experiments^35,36^. This is further supported by the data in Fig. 3b, where the steady-state value is probed for a larger range of tilt values, even reproducing the non-monotonous behavior that is found for small values of the tilt. Note, that the vanishing imbalance, as observed for Δ_↓_ ≈ 1.5*J* (dashed line in Fig. 3b), does not indicate delocalization. It results from localized Wannier-Stark orbitals with equal weight on even and odd sites.

In the presence of weak interactions localization was predicted to survive in the limit of small additional disorder or harmonic confinement, signaled by a finite steady-state imbalance^30,31^. Here, we find that after a small decay at intermediate times a plateau of the imbalance develops, which persists for long evolution times up to 700*τ* (Fig. 3a) in the strongly-interacting regime. A comparison with ED simulations (inset Fig. 3a) in a clean system without spin-dependent tilt and without harmonic confinement for a Néel-ordered initial CDW (as opposed to the random-spin initial state realized in the experiment) further highlights that this non-ergodic behavior is not due to experimental imperfections at least for the experimentally relevant observation times (see Supplementary Fig. 5 for a systematic finite-size scaling analysis). Moreover, this robust steady-state value survives over a wide range of parameters (Fig. 3b). As a function of the tilt it qualitatively follows the behavior of the non-interacting system, but shows consistently lower steady-state values.

The persistence of non-ergodicity down to very small values of the tilt is surprising at first sight. One may expect that for large Bloch-oscillation amplitudes the interactions between particles result in a dephasing of the coherent dynamics that give rise to Wannier-Stark localization in the non-interacting limit and hence cause ergodic behavior^35,36,40–43^. We study the plateau value for Δ_↓_ = 1.1*J* and find that it is largely independent of interactions (Fig. 3c). In a numerical analysis of this regime for a Néel-ordered singlon CDW we indeed find that the imbalance decays to zero for evolution times on the order of 10^4^ *τ* (Supplementary Fig. 5), which further agrees with the finite imbalance measured at ~ 200 *τ*. The observed inversion of the spin-resolved imbalance ${{{{\mathcal{I}}}}}^{\downarrow }\; < \;{{{{\mathcal{I}}}}}^{\uparrow }$ after long evolution times (although Δ_↓_ > Δ_↑_) is explained by the non-monotonic dependence of the stationary imbalance on the tilt for Δ_*σ*_ < 2*J* as shown in Fig. 3b.

For intermediate values of the tilt Δ/*J* ≃ 3 on the other hand, we find a surprisingly robust steady-state imbalance, in agreement with numerical calculations, with a clear interaction dependence (Fig. 3d). The behavior is similar for both spin components and well reproduced by numerical simulations. The deviation between experiment and numerical simulations at larger interaction strengths is most likely due to the finite coupling between 1D chains, which plays a larger role for increased interactions^49^. The steady-state imbalance is symmetric around *U* = 0 due to a dynamical symmetry [for (Δ_↓_ − Δ_↑_) ≪ *J*] between attractive and repulsive interactions (Supplementary Note 2), similar to the homogeneous Fermi-Hubbard model^50,51^. The curve displays a global minimum for intermediate interactions, which we identify with resonant processes at ∣*U*∣ ≃ 2Δ, where two singlons separated by two lattice sites form a doublon. This coincides with regime ① in Fig. 1c, where the largest connectivities were found. The precise value of the resonance is slightly shifted, ${U}_{{{{\rm{res}}}}}\simeq 2{{\Delta }}-8{J}^{2}/(3{{\Delta }})$, due to perturbative corrections for finite *J*/Δ, in agreement with our data (dashed line in Fig. 3e). For large interactions and weak spin-dependence (Δ_↓_ − Δ_↑_) ≪ *J*, we expect the system to recover the non-interacting regime (Supplementary Note 3).

In order to gain additional insights into the observed non-ergodic behavior, we study the properties of our model perturbatively in the large tilt limit for the two distinct regimes ① and ② (Fig. 1c). In regime ②, Δ ≫ *J*, ∣*U*∣, an effective Hamiltonian can be derived in powers of *λ* = *J*/Δ. As predicted^19,20,22,31,33^, we find an emergent dipole-conserving Hamiltonian ${\hat{H}}_{{{{\rm{eff}}}}}^{{{{\rm{dip}}}}}$ [Supplementary Eq. (9)] up to third order in *λ* (Supplementary Note 3), where the dipole-moment operator is defined as ${\sum }_{i,\sigma }i{\hat{n}}_{i,\sigma }$. The dominant off-diagonal terms of ${\hat{H}}_{{{{\rm{eff}}}}}^{{{{\rm{dip}}}}}$ are of similar nature as those in the fragmented Hamiltonians studied previously^19,20^, seemingly consistent with the observed non-ergodic behavior. Yet, higher-order processes ${{{\mathcal{O}}}}({\lambda }^{4})$, relevant for Δ ≃ 3*J*, are expected to melt the CDW within the experimentally studied timescales^19^. These higher-order processes as well as the dominant off-diagonal contribution, however, require the production of doublons, which is penalized by the on-site interaction *U*. We numerically show that this leads to a significant slowdown of the dynamics (Supplementary Note 5), which explains the robustness of the steady-state value observed in the experiment. Thus, for large values of the tilt, the doublon number is effectively conserved as well, as suggested in Ref. ^31^.

**Fig. 4:**
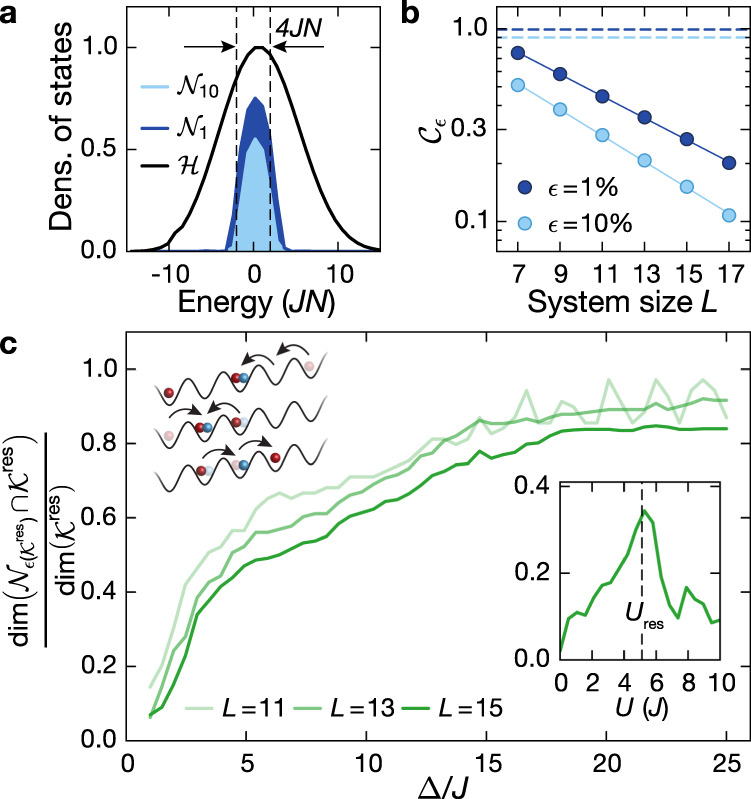
Theoretical analysis of the relevant many-body states for *ω*_*h*_ = 0, Δ_↑_ = Δ_↓_ ≡ Δ and a Néel-ordered initial state. **a** Density of states in the full Hilbert space ${{{\mathcal{H}}}}$ restricted to quarter filling and zero magnetization for the numerical fragments ${{{{\mathcal{N}}}}}_{1}$ (*ϵ* = 1%), ${{{{\mathcal{N}}}}}_{10}$ (*ϵ* = 10%), *U* = 5*J*, Δ = 3*J* and ${T}_{{{{\mathcal{N}}}}}=1000\tau$, normalized to the maximum in ${{{\mathcal{H}}}}$; *L* = 15. **b** Scaling of the finite-time connectivity ${{{{\mathcal{C}}}}}_{\epsilon }$ with system size for a time window ${T}_{{{{\mathcal{N}}}}}=1000\tau$, *U* = 5*J* and Δ = 3*J*. Solid lines are exponential fits to the data. Dashed lines are the prediction for the finite-time connectivity of a thermal state, showing a constant scaling at 1 − *ϵ*. **c** Normalized intersection for *U* = *U*_res_ between the Krylov subspace ${{{{\mathcal{K}}}}}^{{{{\rm{res}}}}}$ and the numerical fragment ${{{{\mathcal{N}}}}}_{\epsilon ({{{{\mathcal{K}}}}}^{{{{\rm{res}}}}})}$ (Methods), where $\,{{\mbox{dim}}}\,({{{{\mathcal{N}}}}}_{\epsilon ({{{{\mathcal{K}}}}}^{{{{\rm{res}}}}})})=\,{{\mbox{dim}}}\,({{{{\mathcal{K}}}}}^{{{{\rm{res}}}}})$ (main text). The schematic shows the most important processes, connecting the states within the Krylov subspace ${{{{\mathcal{K}}}}}^{{{{\rm{res}}}}}$ (Supplementary Note 4). Inset: Normalized intersection as in the main plot for Δ = 3*J*. The dashed line illustrates the resonance condition in regime ①.

On resonance, ∣*U*∣ ≃ 2Δ (regime ① in Fig. 1c), doublons can be formed without energy penalties, possibly leading to faster dynamics. Indeed, after an initial faster dynamics, we find a lower steady-state imbalance, which cannot be solely explained by the second-order resonant tunneling process shown in Fig. 1c, because it leaves the imbalance invariant. In this regime, we derive an effective Hamiltonian ${\hat{H}}_{\,{{\mbox{eff}}}\,}^{{{{\rm{res}}}}}$ [Supplementary Eq. (19)] up to second order in *λ* (the third order vanishes), conserving the dipole moment, the doublon number or the sum of the two (${\sum }_{i,\sigma }i{\hat{n}}_{i,\sigma }+2{\sum }_{i}{\hat{n}}_{i,\uparrow }{\hat{n}}_{i,\downarrow }$). The corresponding symmetry sector exhibits strong fragmentation and results in a finite steady-state imbalance (Supplementary Note 4). In Fig. 4c we show the dominant second-order tunneling terms for our initial state, illustrating the importance of doublon-assisted tunneling processes for the reduction of the steady-state imbalance. For finite *λ* or longer evolution times, higher-order hopping processes ${{{\mathcal{O}}}}({\lambda }^{4})$ enable additional dynamics. These processes are expected to eventually melt the CDW completely, although the required timescales may be very large. In the experiment, we find robust steady-state values even for rather low values of the tilt (Δ ≃ 3*J*) up to evolution times of about 700*τ* (Fig. 3a).

In order to connect the large-tilt limit described by ${\hat{H}}_{\,{{\mbox{eff}}}\,}^{{{{\rm{res}}}}}$ to the experimental parameter regime, we investigate the states within the explored subspace ${{{{\mathcal{N}}}}}_{\epsilon }$, which we denote numerical fragment in analogy to the phenomenon of Hilbert-space fragmentation. For simplicity, we study a clean system (*ω*_*h*_ = 0, Δ_↑_ = Δ_↓_ ≡ Δ) and a Néel-ordered CDW initial state. In Fig. 4a, we show the density of states in the Hilbert space ${{{\mathcal{H}}}}$ and compare it to the density of states in the numerical fragment ${{{{\mathcal{N}}}}}_{\epsilon }$ for different values of the cut-off *ϵ*. Centered around the energy of the initial state, the density of states acquires a finite width within the numerical fragments, that is approximately set by the many-body bandwidth ± 2*JN* (dashed line in Fig. 4a), where *N* = *N*^↑^ + *N*^↓^ denotes the total number of atoms. In stark contrast to thermal systems, the low finite-time connectivity indicates that only a small number of states is relevant for the dynamics. Moreover, it vanishes exponentially in the thermodynamic limit for finite evolution times up to 1000 *τ* (Fig. 4b). Since the perturbative Hamiltonian ${\hat{H}}_{\,{{\mbox{eff}}}\,}^{{{{\rm{res}}}}}$ is only valid in the limit of large tilts, the intersection between the numerically constructed fragment and the analytical one ${{{{\mathcal{K}}}}}^{{{{\rm{res}}}}}$ (“Methods”), which was derived using the perturbative Hamiltonian ${\hat{H}}_{\,{{\mbox{eff}}}\,}^{{{{\rm{res}}}}}$ up to third order in *λ* = *J*/Δ, is small for our experimental parameters Δ = 3*J* and *U* = 5*J* (Fig. 4c). We expect, however, that the two subsectors coincide for *λ* → 0. Indeed the normalized intersection saturates to one, although only for Δ/*J* ≫ 20. For this comparison the cut-off value $\epsilon ({{{{\mathcal{K}}}}}^{{{{\rm{res}}}}})$ is chosen such that $\,{{\mbox{dim}}}\,({{{{\mathcal{N}}}}}_{\epsilon ({{{{\mathcal{K}}}}}^{{{{\rm{res}}}}})})=\,{{\mbox{dim}}}\,({{{{\mathcal{K}}}}}^{{{{\rm{res}}}}})$, since generally, ${{{{\mathcal{N}}}}}_{\epsilon }$ contains a much larger number of states. Despite the large value of *λ* realized in the experiment, we find strong evidence that the slow dynamics is due to kinetic constraints and that the energetically allowed microscopic processes give rise to the phenomenon of Hilbert-space fragmentation in the large tilt limit, as demonstrated for the two regimes (① and ②). This is further supported by the resonance feature that is shown in the inset of Fig. 4c for the resonant regime ①.

## Discussion

In conclusion, we have demonstrated both experimentally and numerically non-ergodic behavior in the tilted 1D Fermi-Hubbard model over a wide range of parameters and have provided a microscopic understanding based on perturbative analytical calculations. For future studies it would be interesting to study the limit of large tilts, where strongly-fragmented effective Hamiltonians were identified and to investigate the initial-state dependence of the transient dynamics. This is a characteristic feature of Hilbert-space fragmentation, where distinct thermalization properties are expected for different fragments^19,20,22^. Although experimentally challenging due to finite evolution times, it would be interesting to reconcile the phenomenon of Stark MBL and Hilbert-space fragmentation, by studying the impact of weak disorder or residual harmonic confinement on the long-time dynamics^33^. Adding periodic modulation as an additional ingredient, other strongly-fragmented models, scarred models and time crystals could be engineered^52–54^ or drive-induced localization could be investigated^55,56^. By tuning the direction of the tilt in a 2D lattice, dipole- and higher-moment conserving models could be realized^20,57^ enabling studies beyond the hydrodynamic regime^58^. Moreover, it will be interesting to explore the connection between lattice gauge theories and the phenomenon of Hilbert-space fragmentation^21,27,29,59–61^, which could be addressed experimentally in a similar model^62^.

## Methods

### Experimental sequence

Our sequence begins with loading a degenerate Fermi gas with temperature *T*/*T*_*F*_ = 0.15(1), where *T*_*F*_ is the Fermi temperature, into a three-dimensional (3D) optical lattice. The wavelength is *λ*_*l*_ = 1064 nm along the *x* direction and *λ*_⊥_ = 738 nm in the transverse directions. Repulsive interactions during loading in combination with a short, off-resonant light pulse after loading ensure an initial state free of double occupancies (Supplementary Note 7). By adding a short lattice with wavelength *λ*_*s*_ = *λ*_*l*_/2 along the *x* direction, we generate a CDW initial state consisting of singlons (Supplementary Note 7). Holding the gas in this deep 3D lattice with a tilted, bichromatic superlattice along the *x* direction, dephases remaining correlations between neighboring sites and suppresses any residual dynamics, while ramping up a magnetic field gradient and adjusting the interaction strength. The lattice depths are 18 *E*_*rs*_ for the short lattice, 20 *E*_*rl*_ for the long lattice and 55 *E*_*r*⊥_ for the transverse lattices. The depths are given in the respective recoil energies, ${E}_{rj}={\hslash }^{2}{k}_{j}^{2}/(2m)$, with *j* ∈ {*l*, *s*, ⊥}, *k*_*j*_ = 2*π*/*λ*_*j*_ the corresponding wave vector, *m* the mass of ^40^K and and *ℏ* = *h*/(2*π*) the reduced Planck constant. The deep transverse lattices decouple the 1D chains aligned along *x* and generate a 2D array of nearly independent 1D systems. The residual coupling along the transverse directions is typically less then 0.03 % of the coupling *J* along *x*. The dynamics to probe the tilted 1D Fermi-Hubbard model described by the Hamiltonian in Eq. (1) is initiated by suddenly switching off the long lattice and quenching the short lattice to depths between 6 *E*_*rs*_ and 8 *E*_*rs*_. Simultaneously, the strength of the dipole trap is adjusted in order to compensate the anti-confining harmonic potential introduced by the lattice (Supplementary Note 8). After a variable evolution time *t* the on-site population is frozen by suddenly ramping up the longitudinal lattices to 18 *E*_*rs*_ and 20 *E*_*rl*_ respectively. Subsequently, we extract the spin-resolved imbalance ${{{{\mathcal{I}}}}}^{\sigma }$, by using a bandmapping technique^63,64^ in conjunction with Stern-Gerlach resolved absorption imaging. Note, that the imbalance is defined as a charge imbalance between even and odd lattice sites in our system and does not probe spin imbalances. The magnetization of the systems is conserved during the evolution and it is equal to zero at all times.

### Initial state

The initial state in all experiments consists of a CDW of singlons, where $\left|\uparrow \right\rangle$ and $\left|\downarrow \right\rangle$ states are randomly distributed on even lattice sites and odd lattice sites are empty. We work with an equal mixture of both states (*N*_↑_ = *N*_↓_) such that the total magnetization is zero. The fraction of residual holes on even lattice sites, due to imperfections in the loading sequence and due to removed doublons is expected to be about 10%^65^. Excellent agreement between the data and numerical simulations, which do not consider residual holes on even sites, indicates, that the hole fraction has a negligible effect on our dynamics. The initial state can be modelled as incoherent mixture within the zero magnetization sector with density matrix $\hat{\rho }=\frac{1}{{{{\mathcal{N}}}}}{\sum }_{\{\sigma \}| {\sum }_{i}{\sigma }_{i} = 0}\left|{\psi }_{0}(\{\sigma \})\right\rangle \left\langle {\psi }_{0}(\{\sigma \})\right|$, where each product state $\left|{\psi }_{0}(\{\sigma \})\right\rangle$, is given by a CDW of singlons and where the sum runs over all ${{{\mathcal{N}}}}$ possible permutations of spin configurations {*σ*}. The product state $\left|{\psi }_{0}(\{\sigma \})\right\rangle$ is defined as $\left|{\psi }_{0}(\{\sigma \})\right\rangle ={\prod }_{i = {{\mbox{even}}}\in {{{\rm{trap}}}}}{\left({\hat{c}}_{i\uparrow }^{{\dagger} }\right)}^{{n}_{i\uparrow }}{\left({\hat{c}}_{i\downarrow }^{{\dagger} }\right)}^{{n}_{i\downarrow }}\left|0\right\rangle$, where ${\hat{c}}_{i\sigma }^{{\dagger} }$ is the fermionic creation operator, *n*_*iσ*_ ∈ {0, 1}, *σ* ∈ {↑, ↓}, *n*_*i*_ = *n*_*i*↑_ + *n*_*i*↓_ ≤ 1 and *i* is the lattice-site index along *x*.

Note, that in the clean translationally-invariant Fermi-Hubbard model the initial CDW corresponds to an infinite temperature state and a finite imbalance value is a hallmark signature of localization. In the tilted model, the spectrum is superextensive complicating a meaningful definition of temperature. This is overcome by transforming into the interaction picture with respect to the tilt potential, which leaves all density observables invariant and allows us to establish the imbalance as a good probe for ergodicity breaking (Supplementary Note 1)

### Details of numerical calculations

The numerical computations that are compared with the experiment in Figs. 2 and 3 of the main text were performed using ED or TEBD. The parameters *J*, Δ_↑_ and Δ_↓_ used in the computations were obtained as fit parameters from the corresponding non-interacting data. Additionally, the effect of harmonic confinement present in the experiment was simulated by scaling the trap frequency by a factor $\sqrt{\frac{{L}_{\exp }}{L}}$ where ${L}_{\exp }=290$ is the system size in the experiment and *L* is the system size used in the numerical calculation. This is done to appropriately simulate the collapse and revival dynamics in the Bloch oscillations induced by the harmonic confinement (Supplementary Note 11).

We use TEBD for short-time dynamics (Fig. 2 of the main text) and ED for long-time dynamics (Fig. 3 of the main text). In ED, we consider the Hilbert space as a tensor product ${{{{\mathcal{H}}}}}_{\uparrow }\otimes {{{{\mathcal{H}}}}}_{\downarrow }$ where ${{{{\mathcal{H}}}}}_{\sigma }$ is the Hilbert space of spin-*σ* atoms. In order to efficiently compute the time dynamics, we decompose each time step in the dynamics into three unitary propagators. One each corresponding to the hopping of the two spin components and the third one corresponding to the on-site potential and interactions. We use a Trotter-Suzuki approximation in this decomposition (see Supplementary Note 13 for details and error analysis). In Fig. 3a, d, we use *L* = 16, *N*_↑_ = *N*_↓_ = 4. In order to effectively model a mixed CDW initial state, in Fig. 3a, this computation is averaged over 20 randomly chosen pure CDW states. In Fig. 3d we use a superposition of pure CDW product states as we are concerned only with time-averaged steady-state value. The parameters *J*, Δ_*σ*_ and the harmonic confinement are fixed by fitting to the corresponding non-interacting data.

In Fig. 2, we use TEBD calculations with *L* = 100 and bond-dimension *χ* = 120. The truncation error was less than 10^−2^. In Fig. 2b, c, we compare the experimental and numerical data in Fourier space. If the two data sets have different number of samplings in the time domain, we scale the numerical data appropriately after the fast Fourier transform.

### Construction of the Krylov subspace

The Krylov subspace (corresponding to the fragment ${{{{\mathcal{K}}}}}^{{{{\rm{res}}}}}$) is constructed by using the effective Hamiltonian on resonance ${\hat{H}}_{{{{\rm{eff}}}}}^{{{{\rm{res}}}}}$ in Supplementary Eq. (19). This Hamiltonian is then interpreted as an adjacency matrix in the Wannier basis and the Krylov subspace consists of all states, which are connected to the Néel-ordered CDW initial state. The Krylov subspace ${{{{\mathcal{K}}}}}^{{{{\rm{res}}}}}$ is closed under time-evolution generated by the effective Hamiltonian ${\hat{H}}_{{{{\rm{eff}}}}}^{{{{\rm{res}}}}}$. Starting from initial states within the Krylov subspace ${{{{\mathcal{K}}}}}^{{{{\rm{res}}}}}$ and including higher-order terms ${{{\mathcal{O}}}}({\lambda }^{4})$, the dynamics is captured only approximately (Supplementary Note 4). An improvement is obtained by further rotating the diagonal basis in which the effective Hamiltonian becomes fragmented with the unitary transformation obtained in powers of *λ* (as given by the Schrieffer-Wolff perturbative expansion (Supplementary Note 3)). This results in a rotated Krylov subspace.

### Construction of the numerical fragment

We define the numerical fragment ${{{{\mathcal{N}}}}}_{\epsilon }$ as the span of a subset ${{{{\mathcal{B}}}}}_{\epsilon }$ of the number basis ${{{\mathcal{B}}}}$ of ${{{\mathcal{H}}}}$, where ${{{\mathcal{H}}}}$ is restricted to quarter filling and zero magnetization. We define the set ${{{{\mathcal{B}}}}}_{\epsilon }$ via its complement, ${{{{\mathcal{B}}}}}_{\epsilon }={{{\mathcal{B}}}}\backslash {{{{\mathcal{B}}}}}_{\epsilon }^{c}$, where ${{{{\mathcal{B}}}}}_{\epsilon }^{c}$ would be ideally defined as the largest subset of ${{{\mathcal{B}}}}$ satisfying ${\max }_{t\,{ < }\,{T}_{{{{\mathcal{N}}}}}}{\sum }_{{n}^{c}\in {{{{\mathcal{B}}}}}_{\epsilon }^{c}}{\left|\left\langle {n}^{c}| \psi (t)\right\rangle \right|}^{2}\, < \,\epsilon$. Here ${T}_{{{{\mathcal{N}}}}}$ defines a time window for the evolution of the initial state $\left|\psi (t=0)\right\rangle$. Equivalently, one could define the subset ${{{{\mathcal{B}}}}}_{\epsilon }$ as the smallest one, satisfying $\mathop{\min }\limits_{t < {T}_{{{{\mathcal{N}}}}}}{\sum }_{n\in {{{{\mathcal{B}}}}}_{\epsilon }}{\left|\left\langle n| \psi (t)\right\rangle \right|}^{2}\ge 1-\epsilon$. We work with the complement, because it is easier to implement numerically. This inequality condition for the complement would ensure that the residual overlap of $\left|\psi (t)\right\rangle$ outside of ${{{{\mathcal{N}}}}}_{\epsilon }$ at any time $t\le {T}_{{{{\mathcal{N}}}}}$ is bounded by *ϵ*. Constructing this ${{{{\mathcal{B}}}}}_{\epsilon }^{c}$, however, involves a search in the powerset of ${{{\mathcal{B}}}}$, which is exponential in the dimension of ${{{\mathcal{H}}}}$. This is intractable even for relatively small system sizes such as *L* = 7. It follows from the inequality ${\max }_{t\,{ < }\,{T}_{{{{\mathcal{N}}}}}}{\sum }_{{n}^{c}}{\left|\left\langle {n}^{c}| \psi (t)\right\rangle \right|}^{2}\le {\sum }_{{n}^{c}}{\max }_{t\,{ < }\,{T}_{{{{\mathcal{N}}}}}}{\left|\left\langle {n}^{c}| \psi (t)\right\rangle \right|}^{2}$ that keeping the latter sum smaller than *ϵ* will ensure that the former sum is also bounded by *ϵ*. Moreover, the latter sum is computationally easier to handle and therefore, we use it to define the fragment. We construct the numerical fragment ${{{{\mathcal{N}}}}}_{\epsilon }$ using a ${{{{\mathcal{B}}}}}_{\epsilon }^{c}$, defined such that ${\sum }_{{n}^{c}}{\max }_{t\,{ < }\,{T}_{{{{\mathcal{N}}}}}}{\left|\left\langle {n}^{c}| \psi (t)\right\rangle \right|}^{2}\, < \,\epsilon$. The gap in the inequality ${\max }_{t\,{ < }\,{T}_{{{{\mathcal{N}}}}}}{\sum }_{{n}^{c}}{\left|\left\langle {n}^{c}| \psi (t)\right\rangle \right|}^{2}\le {\sum }_{{n}^{c}}{\max }_{t\,{ < }\,{T}_{{{{\mathcal{N}}}}}}{\left|\left\langle {n}^{c}| \psi (t)\right\rangle \right|}^{2}$ loosely depends on the sum ${\sum }_{n\in {{{\mathcal{B}}}}}{\max }_{t\,{ < }\,{T}_{{{{\mathcal{N}}}}}}{\left|\left\langle n| \psi (t)\right\rangle \right|}^{2}$, which is in general, not normalized. Although this sum can be as large as the dimension of ${{{\mathcal{H}}}}$, in the examples that we study, it remains small, i.e., < 10 for *L* < 20, and grows logarithmically in the dimension of ${{{\mathcal{H}}}}$.

## Supplementary information

Supplementary Information

## Data Availability

All data files are available from the corresponding author upon reasonable request. Source data are provided with this paper.

## Source data

Source data

## Code availability

The code that supports the plots within this paper are available from the corresponding author upon reasonable request.
