# Supplementary material for: Observing non-ergodicity due to kinetic constraints in tilted Fermi-Hubbard chains
Source: Nat Commun. 2021 Jul 23;12:4490. doi: 10.1038/s41467-021-24726-0 (PMC8302618; doi:10.1038/s41467-021-24726-0)
Supplement: Supplementary file 1 — Supplementary Information [file 41467_2021_24726_MOESM1_ESM.pdf]

Observing non-ergodicity due to kinetic constraints in tilted Fermi-Hubbard chains

Sebastian Scherg[1,2,3], Thomas Kohlert[1,2,3], Pablo Sala[3,4], Frank Pollmann[3,4], Bharath H. M.[1,2,3], Immanuel Bloch[1,2,3], Monika Aidelsburger[1,3*]
[1] *Fakultät für Physik, Ludwig-Maximilians-Universität München, 80799 Munich, Germany*
[2] *Max-Planck-Institut für Quantenoptik, 85748 Garching, Germany*
[3] *Munich Center for Quantum Science and Technology (MCQST), 80799 München, Germany*
[4] *Department of Physics, Technical University of Munich, 85748 Garching, Germany and*
[*] *email: monika.aidelsburger@physik.uni-muenchen.de*

TABLE OF CONTENTS

Supplementary Note 1: Interaction picture 1

Supplementary Note 2: Dynamical symmetry 2

Supplementary Note 3: Effective Hamiltonians 2
- Large tilt limit: dipole conservation 2
- Large interaction limit 3
- Resonant regime $|U| \simeq 2\Delta$ 4

Supplementary Note 4: Fragmentation in the resonant regime $|U| \simeq 2\Delta$ 4
- Renormalized Fermi-Hubbard interaction 4
- Strong Fragmentation 5
- Numerical fragment $\mathcal{N}_\epsilon$ versus fragment $\mathcal{K}^{\text{res}}$ 6

Supplementary Note 5: Constrained dynamics in the presence of higher order terms and on-site interactions 6

Supplementary Note 6: Scaling analysis of the steady-state imbalance and the entanglement entropy 7

Supplementary Note 7: Experimental sequence 8
- General description 8
- CDW preparation and spin-resolved imbalance readout 9

Supplementary Note 8: Creating a homogeneous potential 10

Supplementary Note 9: Creating a linear potential 10

Supplementary Note 10: Interaction averaging 11

Supplementary Note 11: Calibration of parameters 11
- Curvature α and trap frequency ω_h 11
- Spin-dependent tilt Δ_σ 12
- Lattice depth 13
- Onsite interaction U 13

Supplementary Note 12: Extraction of the interaction energy U_{res} 13

Supplementary Note 13: Details on numerical methods 13
- Basis construction 14
- Intermediate time evolution 14
- Error estimates 15
- Long-time evolution 15

References 17

SUPPLEMENTARY NOTE 1: INTERACTION PICTURE

The Hamiltonian in Eq. (1) in the main text does not commute with spatial translations because of the tilted field. However, due to the gauge covariance of the Schrödinger equation, we can transform from the Schrödinger picture to the interaction picture, where the Hamiltonian becomes translational invariant and time-periodic [1, 2]. We apply a unitary transformation $\hat{T}(t) \equiv e^{it\hat{H}_0}$ with $\hat{H}_0 = \sum_\sigma \Delta_\sigma \sum_i i\hat{n}_{i,\sigma}$ according to (we set $\hbar = 1$ in the following)

$$\hat{H}_I(t) = \hat{T}(t)\hat{H}\hat{T}^\dagger(t) - i\hat{T}(t)\partial_t\hat{T}^\dagger(t), \tag{1}$$

with

$$\begin{aligned}\hat{H}_I(t) = &- J\sum_{i,\sigma}\left(e^{-i\Delta_\sigma t}\hat{c}^\dagger_{i,\sigma}\hat{c}_{i+1,\sigma} + \text{h.c.}\right) \\ &+ U\sum_i \hat{n}_{i,\uparrow}\hat{n}_{i,\downarrow},\end{aligned} \tag{2}$$

which for incommensurate $\Delta_\uparrow \neq \Delta_\downarrow$ gives rise to a quasi-periodic Hamiltonian [3]. Since density operators are gauge invariant $\hat{n}_{i,I}(t) = \hat{T}(t)\hat{n}_i\hat{T}^\dagger(t) = \hat{n}_i$, we can study the evolution and long-time value of the imbalance in the interaction picture, starting from the experimentally prepared charge-density wave (CDW) configuration without requiring an additional change of frame. We emphasize that the Hamiltonian in the interaction picture [Supplementary Eq. (2)] explicitly commutes with lattice translations, is well-defined in the thermodynamic limit [2, 4] and avoids the superextensive-scaling contribution of the potential energy to the total energy of the system.

For $\Delta_\uparrow = \Delta_\downarrow \equiv \Delta$, the interacting Hamiltonian [Supplementary Eq. (2)] can be understood as a periodically-driven system [5–7]. For generic ergodic Hamiltonians, we expect the system to heat up to infinite temperature

in the intermediate driving-frequency regime, which we probe in the experiment as a consequence of the energy absorption from the external drive [8, 9]. Therefore, we anticipate the density distribution to become homogeneous and the imbalance to decay to zero at infinite times. In order to give a lower bound on this heating timescale, we can make use of the rigorous theory of prethermalization for periodic [8, 10–13] or quasi-periodic Hamiltonians (for $\Delta_\uparrow \neq \Delta_\downarrow$) [3] in the large tilt $\Delta \gg J$ regime [14]. This predicts a heating timescale $\tau_* \sim \tau e^{c\Delta/\mu}$ for the former, where c is a numerical constant of order one and $\mu \sim (J+U)$. However, for the tilts employed in the experiment $\Delta \sim 3J$, this lower bound is far from the experimentally observed non-ergodicity until times $t \sim 1000\tau$.

SUPPLEMENTARY NOTE 2: DYNAMICAL SYMMETRY

According to the theorem proven in Ref. [15] (Supplementary material section SD), the Fermi-Hubbard model exhibits a dynamical symmetry between repulsive and attractive interactions for any observable, which is invariant under both time-reversal and π-boost $\hat{B}_Q = e^{i\pi\sum_{i,\sigma} i\hat{n}_{i,\sigma}}$, when considering initial states, that are time reversal invariant and only acquire a global phase under the π-boost transformation. While our interaction scans in the main text are consistent with this symmetry, the assumptions are not valid in the presence of a tilt. We can, however, generalize these assumptions and show that the dynamical symmetry holds for our system as well. Under a spatial inversion $\hat{\mathcal{P}}$, i.e. sending $i \to -i$ with respect to the center of a finite chain with length L, the tilted potential of the Hamiltonian changes sign

$$\hat{H}(U,\Delta_\uparrow,\Delta_\downarrow) \xrightarrow{\hat{\mathcal{P}}} \hat{H}(U,-\Delta_\uparrow,-\Delta_\downarrow)\,. \tag{3}$$

Using the π-boost $\hat{B}_Q$ together with the inversion $\hat{\mathcal{P}}$

$$\hat{\mathcal{P}}\hat{B}_Q\hat{H}(U,\Delta_\uparrow,\Delta_\downarrow)\hat{B}_Q^\dagger\hat{\mathcal{P}}^\dagger = -\hat{H}(-U,\Delta_\uparrow,\Delta_\downarrow) \tag{4}$$

an equation similar to Supplementary Eq. (S11) in [15] can be obtained. The experimental observable is the spin-resolved imbalance $\hat{\mathcal{I}}^\sigma = \sum_{i=-\frac{L}{2}}^{\frac{L}{2}}(-1)^i\hat{n}_{i,\sigma}$, which is invariant under inversion $\hat{\mathcal{I}}^\sigma \xrightarrow{\hat{\mathcal{P}}} \hat{\mathcal{I}}^\sigma$ and π-boost $\hat{\mathcal{I}}^\sigma \xrightarrow{\hat{B}_Q} \hat{\mathcal{I}}^\sigma$, but breaks time-reversal symmetry $\hat{\mathcal{T}}$. This symmetry is violated, because the spin degrees of freedom of the density operator $\hat{n}_{i,\sigma}$ are exchanged.

Assuming that $\Delta_\uparrow = \Delta_\downarrow$, the Hamiltonian has an additional SU(2) spin symmetry and is invariant under spin-rotations around $\hat{S}^x = \sum_{\beta,\gamma=\uparrow,\downarrow} 1/2\hat{c}_\beta^\dagger\sigma_{\beta\gamma}^x\hat{c}_\gamma$, where $\sigma_{\beta\gamma}^x$ are the matrix elements of the Pauli matrix. The local observable $\hat{n}_{i,\sigma}$ is invariant under the product of time reversal $\hat{\mathcal{T}}$ and π-rotations around x, and thus we obtain for the time-evolved imbalance operator $\hat{\mathcal{I}}^\sigma_{(U,\Delta_\uparrow,\Delta_\downarrow)}$

$$\begin{aligned}\hat{\mathcal{P}}\hat{B}_Q e^{-i\pi\hat{S}^x}\hat{\mathcal{T}}\hat{\mathcal{I}}^\sigma_{(U,\Delta_\uparrow,\Delta_\downarrow)}(t)\hat{\mathcal{T}}^{-1}e^{i\pi\hat{S}^x}\hat{B}_Q^\dagger\hat{\mathcal{P}}^\dagger =\\ \hat{\mathcal{I}}^\sigma_{(-U,\Delta_\uparrow,\Delta_\downarrow)}(t).\end{aligned} \tag{5}$$

As long as $\Delta_\downarrow - \Delta_\uparrow$ is sufficiently small, an approximate dynamical symmetry is present for our observable.

We next focus on the required symmetries of the initial state. For all experiments, we consider initial states that are an incoherent sum within the zero magnetization sector (thus $N_\uparrow = N_\downarrow$) with density matrix $\hat{\rho} = \frac{1}{N}\sum_{\{\sigma\}|\sum_i\sigma_i=0}|\psi_0(\{\sigma\})\rangle\langle\psi_0(\{\sigma\})|$, where each product state $|\psi_0(\{\sigma\})\rangle$, is given by a CDW of singlons. The sum runs over all possible permutations $\{\sigma\}$ of the spins within the zero magnetization sector. Under the combined action of time reversal and π-rotation around x, this state is left invariant up to a global phase. This is also the case for the π-boost $\hat{B}_Q$. Moreover under spatial inversion $\hat{\mathcal{P}}$ a configuration $\{\sigma_i\}$ is mapped onto another one $\{\sigma_i'\}$ appearing in the mixed state $\hat{\rho}$ with equal weight. Thus, the mixed state is also invariant under $\hat{\mathcal{P}}$. In conclusion, we find for our initial states

$$\mathcal{I}^\sigma_{(U,\Delta_\uparrow,\Delta_\downarrow)}(t) = \mathcal{I}^\sigma_{(-U,\Delta_\uparrow,\Delta_\downarrow)}(t). \tag{6}$$

Note that this dynamical symmetry is weakly broken by experimental imperfections such as the harmonic confinement (see Supplementary Note 9) and varying onsite-interaction strength (see Supplementary Note 10).

SUPPLEMENTARY NOTE 3: EFFECTIVE HAMILTONIANS

In this section we analytically derive several effective Hamiltonians, starting from the clean tilted Fermi-Hubbard model without harmonic confinement and without spin-dependent tilt, described by the Hamiltonian

$$\begin{aligned}\hat{H}_{\mathrm{tFH}} = &-J\sum_{i,\sigma}\left(\hat{c}_{i,\sigma}^\dagger\hat{c}_{i+1,\sigma} + \mathrm{h.c.}\right) + U\sum_i\hat{n}_{i,\uparrow}\hat{n}_{i,\downarrow}\\ &+\Delta\sum_{i,\sigma}i\hat{n}_{i,\sigma}\,.\end{aligned} \tag{7}$$

In particular we will derive effective Hamiltonians corresponding to: (1) large tilt $\Delta \gg J, |U|$, (2) large interaction $|U| \gg J, \Delta$ and (3) the resonant regime $|U| \simeq 2\Delta$.

Large tilt limit: dipole conservation

Here, we focus on the parameter regime $\Delta \gg |U|\,, J$ and derive an effective Hamiltonian using the high-frequency expansion (HFE) in the interaction picture.

The Hamiltonian in the interaction picture is time-periodic $\hat{H}_I(t+\frac{2\pi}{\Delta}) = \hat{H}_I(t)$. According to Floquet theory [6, 7] the unitary evolution generated by $\hat{H}_I(t)$ can be written as

$$\hat{U}_I(t,t_0) = e^{-i\hat{K}_{\text{eff}}(t)} e^{-i(t-t_0)\hat{H}_{\text{eff}}} e^{i\hat{K}_{\text{eff}}(t_0)}, \qquad (8)$$

with a time-independent Floquet-gauge invariant Hamiltonian $\hat{H}_{\text{eff}}$ and a gauge-dependent and time-periodic kick operator $\hat{K}_{\text{eff}}(t)$. It has been noticed that the first orders in the perturbative Schrieffer-Wolff (SW) transformation approach for static Hamiltonians (see e.g., [16]), coincide with those in the HFE in the interaction picture (which provides the gauge-invariant effective Hamiltonian) [7, 17], with the SW generator given by the kick operators. Following this approach, we obtain the effective Hamiltonian as a Floquet expansion in powers of $1/\Delta$ with $\hat{H}_{\text{eff}} = \sum_n \hat{H}_{\text{eff}}^{(n)}$ and $\hat{K}_{\text{eff}}(t) = \sum_n \hat{K}_{\text{eff}}^{(n)}(t)$. Up to third order the effective Hamiltonian is [5–7, 18]:

$$\begin{aligned}\hat{H}_{\text{eff}}^{\text{dip}} =& J^{(3)}\hat{T}_3 + 2J^{(3)}\hat{T}_{XY} + U\Big(1-\frac{4J^2}{\Delta^2}\Big)\sum_i \hat{n}_{i,\uparrow}\hat{n}_{i,\downarrow} \\ &+ 2J^{(3)}\sum_{i,\sigma}\hat{n}_{i,\sigma}\hat{n}_{i+1,\bar{\sigma}}, \end{aligned} \qquad (9)$$

up to constant terms, where $\bar{\sigma} = \{\downarrow,\uparrow\}$ indicates the respective opposite spin of $\sigma = \{\uparrow,\downarrow\}$, $J^{(3)} = \frac{J^2U}{\Delta^2}$ and

$$\hat{T}_3 = \sum_{i,\sigma} \hat{c}_{i,\sigma}\hat{c}^\dagger_{i+1,\sigma}\hat{c}^\dagger_{i+1,\bar{\sigma}}\hat{c}_{i+2,\bar{\sigma}} + \text{h.c.}, \qquad (10)$$

$$\hat{T}_{XY} = \sum_{i,\sigma} \hat{c}^\dagger_{i,\bar{\sigma}}\hat{c}_{i+1,\bar{\sigma}}\hat{c}^\dagger_{i+1,\sigma}\hat{c}_{i,\sigma}. \qquad (11)$$

The kick-operator to third order is expressed as

$$\begin{aligned}\hat{K}_{\text{eff}}(t) =& -i\frac{J}{\Delta}\sum_{i,\sigma}\big(\hat{c}^\dagger_{i,\sigma}e^{-it\Delta}\hat{c}_{i+1,\sigma} - \text{h.c.}\big) \\ &- i\frac{JU}{\Delta^2}\sum_{i,\sigma}\big(\hat{n}_{i+1,\bar{\sigma}} - \hat{n}_{i,\bar{\sigma}}\big)\times \\ &\big(\hat{c}^\dagger_{i,\sigma}e^{-it\Delta}\hat{c}_{i+1,\sigma} - \text{h.c.}\big)\end{aligned} \qquad (12)$$

and the time-evolution operator is approximated as

$$\hat{U}_I(t,t_0) \approx e^{-i\hat{K}_{\text{eff}}(t)} e^{-i(t-t_0)\hat{H}_{\text{eff}}} e^{i\hat{K}_{\text{eff}}(t_0)}. \qquad (13)$$

Rotating back to the Schrödinger picture, we find

$$\begin{aligned}\hat{U}(t,t_0) =& e^{-it\hat{H}_0}\hat{U}_I(t,t_0)e^{it_0\hat{H}_0} \\ &\approx e^{-\hat{S}} e^{-i(t-t_0)\big(\hat{H}_{\text{eff}}+\hat{H}_0\big)} e^{\hat{S}},\end{aligned} \qquad (14)$$

where we have used the fact that $[\hat{H}_{\text{eff}}, \hat{H}_0] = 0$ and that $e^{-it\hat{H}_0}\hat{K}_{\text{eff}}(t)e^{it\hat{H}_0} = \hat{K}_{\text{eff}}(0)$ [17], namely the product on the left hand side does not depend on time. Therefore, the Hamiltonian in the large-tilt limit can be approximated (up to higher-order terms) via

$$\hat{H} \approx e^{-\hat{S}}\big(\hat{H}_{\text{eff}} + \hat{H}_0\big)e^{\hat{S}}, \qquad (15)$$

taking the form of a perturbative SW transformation at third order in J/Δ, with the SW generator given by $\hat{S} = i\hat{K}_{\text{eff}}(0)$. We have thus obtained an effective Hamiltonian which conserves the dipole moment (or center of mass $\sum_{i,\sigma} i\hat{n}_{i,\sigma}$), with $\hat{T}_3$ in Supplementary Eq. (10) the strongly-fragmented dipole-conserving Hamiltonian studied in [19, 20], up to additional spin degrees of freedom. The fact that the hopping rate $J^{(3)}$ is proportional to the interaction strength highlights that interactions are necessary to generate dipole-conserving processes [21] (pure off-diagonal non-interacting contributions destructively interfere at any order). $J^{(3)}$ agrees with the two particle picture [21] yielding $J_{\text{eff}} \propto \frac{UJ^2}{\Delta^2-U^2}$ with $|U| \ll \Delta$. For CDW initial states of singlons, the connected dynamical sector $\mathcal{K}$ only represents a vanishing fraction of the whole (effective) symmetry sector $\mathcal{S}$, thus severely restricting the dynamics of the system. The dipole-conserving processes in general involve the generation of doublons. This is, however, penalized by the Fermi-Hubbard on-site interaction in Supplementary Eq. (9) and therefore, we expect a slowing down of the dipole-conserving dynamics (see Supplementary Note 5). The additional spin-exchange $\hat{T}_{XY}$ increases the connectivity, but cannot fully connect the whole dipole symmetry sector and the system remains fragmented.

Large interaction limit

We study the limit $|U| \gg J, \Delta$ with $\big||U| - n\Delta\big| \neq 0$ for any $n \in \mathbb{N}$ to avoid possible resonances [11]. In this limit, the number of doublons N_{doub} is effectively conserved up to times that scale exponentially in the interaction strength U [22, 23]. Dealing with initial singlon configurations, we have $N_{\text{doub}} = 0$ and assume a negligible fraction of dynamically-generated doublons after the quench. In this limit, the effective Hamiltonian provides non-trivial dynamics at first order in perturbation theory

$$\begin{aligned}\hat{H}^U_{\text{eff}} =& -J\sum_{i,\sigma}\big[(1-\hat{n}_{i,\bar{\sigma}})\hat{c}^\dagger_{i,\sigma}\hat{c}_{i+1,\sigma}(1-\hat{n}_{i+1,\bar{\sigma}}) + \text{h.c.}\big] \\ &+ \Delta\sum_{i,\sigma} i\hat{n}_{i,\sigma}.\end{aligned} \qquad (16)$$

Note that the dynamics generated by this Hamiltonian conserves the configuration of spins $|\{\sigma_1,\ldots,\sigma_N\}\rangle$, with $\sigma_i = \{\uparrow,\downarrow\}$ and the total particle number N. The last term in Supplementary Eq. (16) equally couples to both spin degrees of freedom and the many-body states expressed in the particle-number basis factorize in terms of N free Wannier-Stark localized spinless fermions with many-body wave function $|\{i_1,\ldots,i_N\}\rangle$,

with $i_i \in \{-\frac{L}{2}, \ldots, 0, \ldots, \frac{L}{2}\}$ and fixed spin configuration $|\{\sigma_1, \ldots, \sigma_N\}\rangle$ [24]. As a result the effective Hamiltonian [Supplementary Eq. (16)] takes the form

$$\hat{H}^{U}_{\text{eff}} = -J \sum_i \left(\hat{c}_i^\dagger \hat{c}_{i+1} + \text{h.c.}\right) + \Delta \sum_i i\hat{n}_i. \tag{17}$$

This has to be compared with the non-interacting Hamiltonian in Supplementary Eq. (**??**) for $N = N_\uparrow + N_\downarrow$ spinful fermions, which for a one-body observable like the imbalance gives exactly the same result. Higher-order terms at finite U do not conserve the spin configuration $|\{\sigma_1, \ldots, \sigma_N\}\rangle$. The leading terms in second-order perturbation are spin-exchange and longer-range hopping terms, as well as nearest-neighbors interactions $-2J^2/U \sum_{i,\sigma} \hat{n}_{i,\sigma}\hat{n}_{i+1,\bar{\sigma}}$, which lead to an interaction-induced decay of the imbalance to lower values compared to the non-interacting case at sufficiently long times ($t \sim U/J^2$).

The experimental setup has a weak spin-dependent tilt ($\Delta_\downarrow - \Delta_\uparrow \approx 0.3J < J$), hence, the previous discussion provides a good approximation for sufficiently strong U. Only in the limit $\Delta_\downarrow - \Delta_\uparrow > J$, the effective Hamiltonian in Supplementary Eq. (16) does not map onto spinless fermions, because it depends on the spin configuration. This implies that the non-quadratic interaction terms, appearing in the hopping, have to be taken into account. This corresponds to two Stark ladders with different slopes constraining the mobility within each other.

Resonant regime $|U| \simeq 2\Delta$

The singlon CDW structure of the initial states makes the resonance $|U| \simeq 2\Delta$ more prominent in the dynamics than the one at $|U| = \Delta$, where any hopping process from the initial state would require an energy Δ. Consider the family of states for which $\hat{H}_0 = \Delta \sum_{i,\sigma} i\hat{n}_{i,\sigma} + 2\Delta \sum_i \hat{n}_{i,\uparrow}\hat{n}_{i,\downarrow}$ takes the same value. This defines a subspace, within which an effective Hamiltonian $\hat{H}_{\text{eff}}$ with $[\hat{H}_0, \hat{H}_{\text{eff}}] = 0$ can be obtained as an expansion in $\lambda = J/\Delta$. Such Hamiltonian can either independently conserve the dipole moment and the number of doublons or the sum of the two. Using a Schrieffer-Wolf unitary transformation $e^{\lambda\hat{S}}$ [16, 22, 25] with $\hat{S} = \sum_{n=0} \lambda^n \hat{S}_n$ up to an optimal order n^*, we can generate order-by-order an effective local Hamiltonian that is "close" to a block diagonal form with respect to $\hat{H}_0$

$$e^{\lambda\hat{S}_{n\le n^*}} \hat{H} e^{-\lambda\hat{S}_{n\le n^*}} = \hat{H}^{(n^*)}_{\text{eff}} + \hat{V}_{n\ge n^*}, \tag{18}$$

where $[\hat{H}_0, \hat{V}_{n\ge n^*}] \neq 0$ with $\hat{V}_{n\ge n^*}$ exponentially small in $1/\lambda$ [12, 13, 22]. In particular, we obtain the explicit form of the effective Hamiltonian to second order in λ:

$$\begin{aligned}\hat{H}^{\text{res}}_{\text{eff}} =& \hat{H}_0 + \frac{8J^2}{3\Delta} \sum_i \hat{n}_{i,\uparrow}\hat{n}_{i,\downarrow} - \frac{4J^2}{3\Delta}\hat{T}_{XY} + \frac{4J^2}{3\Delta}\hat{H}_D \\ &+ \frac{J^2}{\Delta}\hat{T}_1 - \frac{2J^2}{\Delta}\hat{T}_2 + \frac{2J^2}{3\Delta}\hat{T}^D_3\ ,\end{aligned} \tag{19}$$

with

$$\begin{aligned}\hat{T}_1 &= \sum_{i,\sigma} (1 - \hat{n}_{i+2,\bar{\sigma}})(1 - 2\hat{n}_{i+1,\bar{\sigma}})\hat{n}_{i,\bar{\sigma}}\hat{c}^\dagger_{i,\sigma}\hat{c}_{i+2,\sigma} + \text{h.c.}, \\ \hat{T}_2 &= \sum_{i,\sigma} (1 - \hat{n}_{i+2,\bar{\sigma}})\hat{n}_{i,\sigma}c^\dagger_{i,\bar{\sigma}}\hat{c}_{i+1,\bar{\sigma}}\hat{c}^\dagger_{i+1,\sigma}\hat{c}_{i+2,\sigma} + \text{h.c.}, \\ \hat{T}^D_3 &= \sum_{i,\sigma} (\hat{n}_{i,\sigma} - \hat{n}_{i+2,\bar{\sigma}})^2(1 - 2(\hat{n}_{i+2,\bar{\sigma}} - \hat{n}_{i,\sigma})) \\ &\times \hat{c}_{i,\bar{\sigma}}\hat{c}^\dagger_{i+1,\bar{\sigma}}\hat{c}^\dagger_{i+1,\sigma}\hat{c}_{i+2,\sigma} + \text{h.c.} \\ \hat{H}_D &= -2\sum_i \hat{n}_{i,\uparrow}\hat{n}_{i,\downarrow}(\hat{n}_{i+1} - \hat{n}_{i-1}) - \sum_{i,\sigma} \hat{n}_{i,\sigma}\hat{n}_{i+1,\bar{\sigma}}.\end{aligned} \tag{20}$$

The first term in the expansion of the SW generator $\hat{S} = \sum \lambda^n \hat{S}_n$ takes the form

$$\hat{S}_0 = \sum_{i,\sigma} \left(1 - 2\hat{n}_{i,\bar{\sigma}} - \frac{2}{3}\hat{n}_{i+1,\bar{\sigma}} + \frac{8}{3}\hat{n}_{i,\bar{\sigma}}\hat{n}_{i+1,\bar{\sigma}}\right)\hat{c}^\dagger_{i+1,\sigma}\hat{c}_{i,\sigma} - \text{h.c.}. \tag{21}$$

Similar to the Hamiltonian $\hat{H}^{\text{dip}}_{\text{eff}}$ [Supplementary Eq. (9)], $\hat{H}^{\text{res}}_{\text{eff}}$ involves a "dressed" $\hat{T}^D_3$ term conserving both the dipole moment and the number of doublons independently, giving rise to doublon-assisted dipole conserving processes. This is the diagonal part of $\hat{T}_3$ commuting with $\hat{N}_{\text{doub}}$.

SUPPLEMENTARY NOTE 4: FRAGMENTATION IN THE RESONANT REGIME $|U| \simeq 2\Delta$

Here, we study the effective Hamiltonian $\hat{H}^{\text{res}}_{\text{eff}}$ in the resonant regime [Supplementary Eq. (19)] regarding both its diagonal and off-diagonal terms in the number basis. While the diagonal terms cause a renormalized Fermi-Hubbard interaction and a shifted resonance, the off-diagonal terms result in a connectivity of the initial product states with other number basis states, causing strong fragmentation and a finite steady-state imbalance.

Renormalized Fermi-Hubbard interaction

The diagonal terms of the effective Hamiltonian in Supplementary Eq. (19) add long-range interactions and renormalize the Fermi-Hubbard interaction such that the resonant point is shifted for finite λ according to $U + \frac{8J^2}{3\Delta} + \mathcal{O}(\frac{J^2}{\Delta}) = 2\Delta$ and the overall resonance is broadened. We numerically identify the resonance for large tilt $\Delta = 10J$ (Supplementary Fig. 1a) and intermediate tilt $\Delta = 3J$ (Supplementary Fig. 1b) using different system sizes $L = 9, 11, 13, 15$ probing the time-averaged imbalance $\bar{\mathcal{I}}(T) \equiv \frac{1}{T}\int_0^T dt\,\mathcal{I}(t)$. Supplementary Fig. 1 depicts a sharp resonance at strong tilt, while a rather broad feature is present at intermediate tilt. Away from $|U| \simeq 2\Delta$ in the large U regime, we find that the system is Wannier-Stark localized. For both regimes, the numerical results

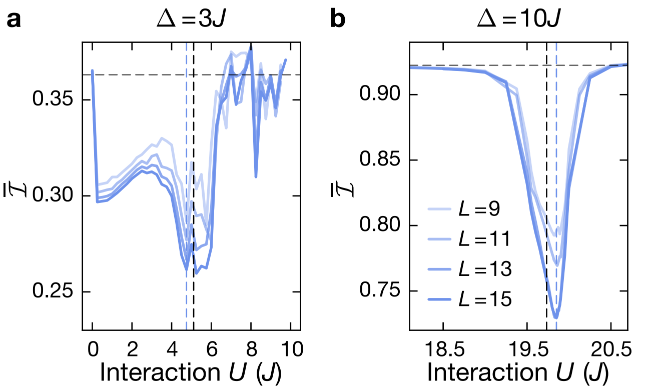

Supplementary Figure 1. **Identification of the resonance** $|U| \simeq 2\Delta$**.** ED calculation of the time-averaged imbalance $\overline{\mathcal{I}} = 1/T\int_0^T \mathcal{I}dt$ for system sizes $L = 9, 11, 13, 15$ with increasing opacity and $T = 1000\tau$. We use $\Delta_\uparrow = \Delta_\downarrow$. The horizontal dashed line shows the analytical value $\mathcal{J}_0(4J/\Delta)^2$ in the non-interacting case ($U = 0$) in the limit $T \to \infty$. The vertical black dashed line indicates the resonant point, including the second order correction $U_{\rm res} = 2\Delta - 8J^2/(3\Delta)$. **a** Time-averaged imbalance for $\Delta = 3J$. Close to the minimum, we use a uniform grid with spacing $\delta U = 0.25J$ and identify the lowest imbalance at $U = 4.75J$ (blue dashed line). **b** Time-averaged imbalance for $\Delta = 10J$. Close to the minimum we use a grid with steps $\delta U = 0.01J$, allowing us to locate the minimum at $U = 19.85J$ (blue dashed line).

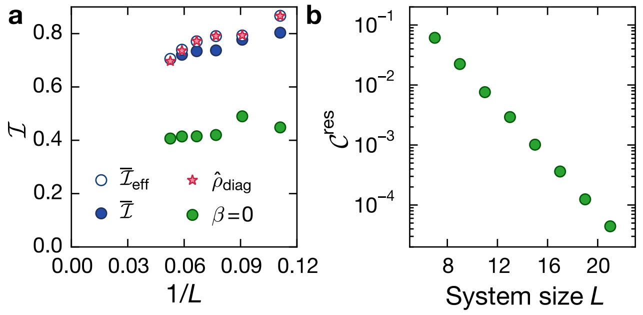

Supplementary Figure 2. **Imbalance in the large tilt limit** $\Delta = 10J$**.** **a** Finite size scaling of the long-time value of the imbalance calculated with the effective Hamiltonian in Supplementary Eq.(19) using a time-averaged imbalance with $T = 3000\tau$ ($\overline{\mathcal{I}}_{\rm eff}$), a diagonal ensemble ansatz ($\hat{\rho}_{\rm diag}$) and an infinite temperature prediction ($\beta = 0$). Additionally, the original Hamiltonian in Supplementary Eq. (7) is used to compare to the time-averaged imbalance calculated with $T = 1000\tau$ ($\overline{\mathcal{I}}$). All ED calculations were done with a Néel-ordered CDW initial state. **b** System size scaling of the connectivity $\mathcal{C}^{\rm res}$ of the fragment $\mathcal{K}^{\rm res}$, capturing the Néel-ordered CDW initial state within the full Hilbert space $\mathcal{H}$, restricted to quarter filling and zero magnetization.

are consistent with the analytic prediction for the shifted resonance to second order even after 1000τ, as shown in Fig. 3e in the main text.

Strong Fragmentation

The off-diagonal terms of the effective Hamiltonian in Supplementary Eq. (19) consist of three different kinds of correlated hoppings $\hat{T}_1, \hat{T}_2, \hat{T}_3^D$ (see Fig. 4d in the main text) and all hopping rates scale as J^2/Δ. Since $[\hat{H}^{\rm res}_{\rm eff}, \hat{H}_0] = 0$, $\hat{H}_0$ becomes a new global quantum number fixed by the initial configuration, i.e. the linear combination of the dipole moment and the number of doublons is perturbatively conserved. Generically, after fixing this new global quantum number, the corresponding symmetry sector $\mathcal{S}$ is fully connected by the action of the effective Hamiltonian and the Krylov subspace, hosting the initial state, agrees with the global symmetry sector $\mathcal{S}$. In contrast, we realize that this is not the case for the effective Hamiltonian in Supplementary Eq. (19). Here, the symmetry sector decomposes into exponentially many disconnected fragments $\mathcal{K}^{\rm res}$ and the initial state remains trapped within such a fragment without exploring the whole symmetry sector.

For the subsequent analysis, we use a Néel-ordered CDW initial state, expected to show the strongest interaction effects and fastest dynamics. The correlated hoppings $\hat{T}_1, \hat{T}_2, \hat{T}_3^D$ of the effective Hamiltonian connect the initial state with a set of states defining the fragment $\mathcal{K}^{\rm res}$. Similarly to the finite-time connectivity $\mathcal{C}_\epsilon$ of the numerical fragment (Fig. 1, Fig. 4 in the main text), we define the connectivity $\mathcal{C}^{\rm res} = \dim(\mathcal{K}^{\rm res})/\dim(\mathcal{H})$ as the ratio between the dimension of the fragment $\dim(\mathcal{K}^{\rm res})$ and the Hilbert space $\dim(\mathcal{H})$, which is restricted to quarter filling and zero magnetization. Experimentally, we do not realize Néel-ordered CDW states, but the connectivity of our initial state with random CDW spin-sector is the same as for the Néel-ordered CDW state. In Supplementary Fig.2b, we show the system size scaling of the connectivity and find that it vanishes exponentially in the thermodynamics limit as expected in the regime of strong fragmentation (The same scaling holds for the connectivity of the fragment within the symmetry sector $\mathcal{S}$.) [19, 20, 26, 27].

In Supplementary Fig. 2a we analyze the system size scaling of both the infinite temperature (within the fragment containing the initial state) and diagonal ensemble predictions for the imbalance, obtaining a positive result in both cases for system sizes $L = 9, 11, 13, 15, 17, 19$ with no clear convergence towards zero imbalance in the thermodynamic limit. The scaling of the infinite temperature prediction suggests a finite value even in this limit. This apparent finite imbalance for $\hat{H}^{\rm res}_{\rm eff}$ could be interpreted as follows: Given an initial state that breaks even-odd sublattice symmetry, most dynamical processes in Supplementary Eq.(19), except those generated by $\hat{T}_3^D$, do only transport particles in one of the sublattices. Thus, most states within the fragment have positive imbalance in agreement with the positive infinite temperature value. This explanation is in line with the observed ergodicity-breaking in dipole-conserving systems, where

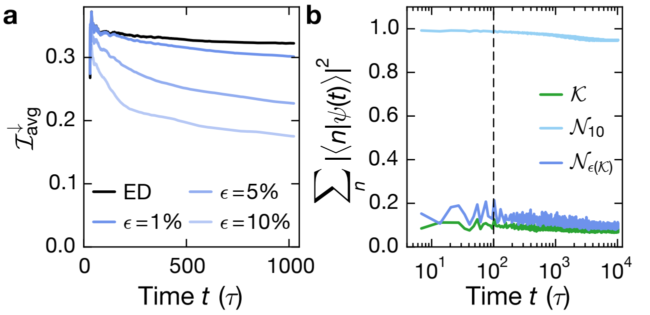

Supplementary Figure 3. **Numerical fragment $\mathcal{N}_\epsilon$.** Both figures use $U = 5J$ and $\Delta_\downarrow = \Delta_\uparrow = 3J$. **a** Imbalance time traces calculated with different sets of states $\mathcal{N}_\epsilon$ and ED for $U = 5J$ and $\Delta = 3J$. $I^{\downarrow}_{\text{avg}}$ is calculated using a cumulative sum to reduce fluctuations; $L = 11$. **b** Contribution of the set of states $|n\rangle$ in the numerical fragments $\mathcal{N}_{\epsilon(\mathcal{K}^{\text{res}})}$ and $\mathcal{N}_{10}$ and the fragment $\mathcal{K}^{\text{res}}$ to the time-evolved initial state $|\psi(t)\rangle$. $T_{\mathcal{N}} = 100\tau$ (dashed line).

a finite value of the autocorrelation was observed even at infinite temperatures [19].

In Supplementary Fig. 2a, simulations with the exact Hamiltonian $\hat{H}_{\text{tFH}}$ [Supplementary Eq. (7)] for $\Delta = 10J$, $U = 19.85J$ agree well with the results of the effective Hamiltonian [Supplementary Eq. (19)] even up to remarkably long times $T \sim 10^3\tau$. Consistent with a perturbative expansion in λ, which neglects higher order terms in the effective Hamiltonian, it yields a systematically larger imbalance compared to the original Hamiltonian. Since the conservation law, i.e. the linear combination of the dipole moment and the number of doublons, only holds perturbatively, one would expect that it is valid only up to a certain timescale.

Numerical fragment $\mathcal{N}_\epsilon$ versus fragment $\mathcal{K}^{\text{res}}$

In Supplementary Fig. 3a, we investigate how well the imbalance can be captured, when using only the states within the numerical fragment. These states correspond to a small fraction compared to the states within the full Hilbert space and this fraction was found to vanish in the thermodynamic limit (Fig ??b). We show imbalance time traces, calculated with a cumulative sum to reduce fluctuations and compare traces with different cut-off values ϵ to the exact numerical result, which we obtained with ED. We find that for $U = 5J$ and $\Delta = 3J$, already with a cut-off $\epsilon = 1\%$ we can reproduce the exact result well, larger cut-off values result in a deviation, which becomes more pronounced at later times. In Supplementary Fig. 3b the overlap of the states $|n\rangle$ in different numerical fragments and in the Krylov subspace $\mathcal{K}^{\text{res}}$ with the time evolved initial state $|\psi(t)\rangle$ is analyzed by calculating $\sum_n |\langle n|\psi(t)\rangle|^2$. While the overlap in our parameter regime is poorly captured by the states in the Krylov subspace $\mathcal{K}^{\text{res}}$ (as expected because these states best describe the time evolution only in the limit $|U| = 2\Delta \gg J$), we can find the proper states by choosing a small enough ϵ. For $\mathcal{N}_1$ we get $\sum_n |\langle n|\psi(t)\rangle|^2 \approx 1$, which shows a very weak decay even up to $t = 10^4\tau$. Note that it is crucial to choose enough states for the numerical fragment. If we choose the same number of states as used in the Krylov subspace $\mathcal{K}^{\text{res}}$ for the numerical fragment $\mathcal{N}_{\epsilon(\mathcal{K}^{\text{res}})}$, we cannot capture the time evolved initial state $|\psi(t)\rangle$ well.

SUPPLEMENTARY NOTE 5: CONSTRAINED DYNAMICS IN THE PRESENCE OF HIGHER ORDER TERMS AND ON-SITE INTERACTIONS

Dynamics caused by fragmentation is captured by effective Hamiltonians and is therefore a transient phenomenon. The perturbative derivation of the effective Hamiltonian neglects higher-order terms which are known to eventually couple different fragments and symmetry sectors, such that the dynamics no longer solely occur within a certain fragment. Estimating the time scales, which capture the dynamics caused due to fragmentation, requires a detailed analysis of both the diagonal and off-diagonal terms of the effective Hamiltonian. Note that the off-diagonal term $\hat{T}_3$, occurring at a rate $J^{(3)} = \frac{J^2 U}{\Delta^2}$ in the dipole conserving limit ($\Delta/J \to \infty$, Supplementary Eq. (9)), requires the production of doublons. Creating a doublon is, however, penalized by the diagonal Fermi-Hubbard interaction with strength $\sim U$. Therefore, an initial state consisting of a CDW of singlons without doublons remains frozen for exponentially long times $t \geq e^{c(\Delta/J)^2}$, analogously to the stability of doublons in the repulsive Fermi-Hubbard model in the $U \gg J$ regime [8, 23]. This effectively gives rise to a fragmentation not only due to the conservation law of the respective effective Hamiltonian, but additionally due to the conservation of the doublon number [27]. A similar argument can be made for the time scale on which higher-order off-diagonal terms, coupling different fragments, become effective and eventually destroy fragmentation. We will give a brief outline here for the dipole conserving limit, where higher order terms are easier to capture. These terms add longer-range processes to the effective Hamiltonian and in general order-n terms generate longer range-n processes whose effective hopping rate scales as $J^{(n)} \sim J^{2k} U^{n-2k}/\Delta^{n-1}$ for some k. Any even order vanishes due to destructive interference: For every process started by a particle hopping to the left, there exists another process with a particle hopping to the right, thus contributing with opposite signs. The hopping rate of the next non-vanishing fifth-order scales as $J^{(5)} \sim J^4 U/\Delta^4$.

Using a qualitative Kato-Bloch perturbative approach [28, 29], which is easier to handle than a Schrieffer-Wolf transformation or a Floquet expansion [30] for higher-order terms, two terms emerge at fifth order in the dipole conserving limit: a 5-local Hamiltonian

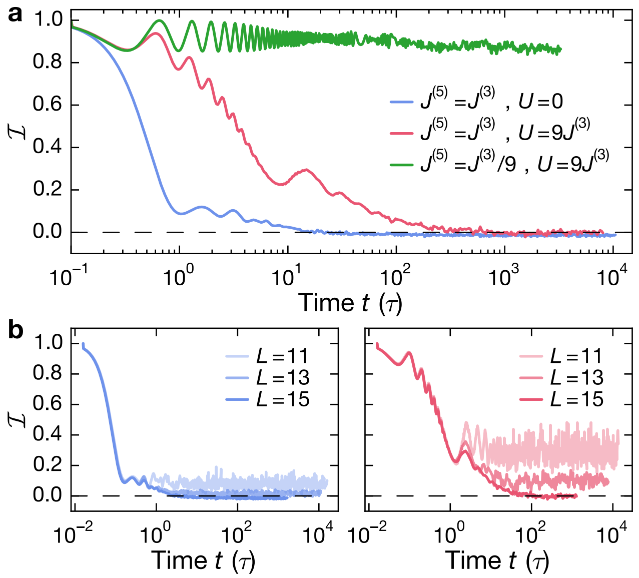

Supplementary Figure 4. **Role of higher-order diagonal and off-diagonal terms.** ED calculation of the imbalance for the Hamiltonian $\hat{H} = J^{(3)}(\hat{T}_3 + \hat{T}_{XY}) + J^{(5)}(\hat{T}_4 + \hat{T}_5) + U\hat{N}_{\text{doub}}$. **a** Imbalance for $J^{(5)} = J^{(3)}$, $U = 0$ (blue), $J^{(5)} = J^{(3)}$, $U = 9J^{(3)}$ (red), and $J^{(5)} = J^{(3)}/9$, $U = 9J^{(3)}$ (green) for system size $L = 15$. **b** Finite size scaling of the imbalance for $J^{(5)} = J^{(3)}$, $U = 0$ (left) and for $J^{(5)} = J^{(3)}$, $U = 9J^{(3)}$ (right). In both cases, we use $L = 11, 13, 15$ and increasing opacity corresponds to increasing system size.

$\hat{T}_5 = \sum_{i,\sigma}\left(\hat{c}_{i,\sigma}\hat{c}^\dagger_{i+2,\sigma}\hat{c}^\dagger_{i+2,\bar{\sigma}}\hat{c}_{i+4,\bar{\sigma}} + \text{h.c.}\right)$, with two opposite spins hopping to an intermediate site, requiring the creation of a doublon in the central site. A 4-local term $\hat{T}_4$ similar to the $\hat{H}_4$ Hamiltonian studied in [19] $\hat{T}_4 = \sum_{i,\sigma}\left(\hat{c}_{i,\sigma}\hat{c}^\dagger_{i+1,\sigma}\hat{c}^\dagger_{i+2,\bar{\sigma}}\hat{c}_{i+3,\bar{\sigma}} + \text{h.c.}\right)$, which populates nearby sites with opposite spin, thus interacting via the nearest-neighbor interaction appearing at third order. We now consider the time-evolution of an Néel-ordered CDW initial state for system size $L = 15$ with the toy model Hamiltonian $\hat{H} = J^{(3)}(\hat{T}_3 + \hat{T}_{XY}) + J^{(5)}(\hat{T}_4 + \hat{T}_5) + U\hat{N}_{\text{doub}}$ using $J^{(3)} = 1$ as unit of energy. Here, $\hat{N}_{\text{doub}}$ measures the number of doublons. In Supplementary Fig. 4a we clearly observe an exponential decay of the imbalance for $J^{(3)}, J^{(5)} \sim O(1)$ and $U = 0$ in agreement with the results in Refs. [19, 20]. The decay time scale increases strikingly, when adding on-site interactions such that $J^{(3)} = J^{(5)} = 1$, $U = 9$ corresponding to a ratio $U/J^{(3)} = 9$ in the perturbative expansion, which is consistent with $\Delta = 3J$ although the higher-order term is still unrealistically large ($J^{(5)} = J^{(3)}$).

A more realistic regime is captured with $J^{(5)} = J^{(3)}/9$ and $U = 9$ due to the perturbative scalings. Here, the imbalance clearly stays finite on our time scales. Thus, the energy penalty given by the on-site interaction has a drastic effect on the decay of the imbalance caused by higher order terms, slowing down the dynamics tremendously. Supplementary Fig. 4b and Supplementary Fig. 4c show a finite-size scaling in the regimes $J^{(5)} = J^{(3)}$ with $U = 0$ and $J^{(3)} = J^{(5)}$ with $U = 9$, clearly indicating that large system sizes are necessary to capture the correct steady-state imbalance.

Unlike the previous regime, at perfect resonance $U_{\text{res}} = 2\Delta \gg J$, neither the lowest-order dynamical processes generated by $\hat{H}^{\text{res}}_{\text{eff}}$ in Supplementary Eq. (19) nor in general higher-order terms, are energetically suppressed. Thus, at a time scale given by the fourth-order term $t \propto \Delta^3/J^4$, fragmentation phenomena are expected to breakdown with the result that imbalance decays. Note that the third-order and in general any odd-order term vanishes due to the CDW initial state, requiring an even number of hoppings for a resonant exchange between tilt and interaction energy. Locating such a resonant point (at finite Δ) requires fine-tuning: every order in perturbation theory gives a diagonal contribution renormalizing the Fermi-Hubbard interaction. As numerically shown in Supplementary Fig. 1a, this is even more subtle at lower values of the tilt. In general, we expect a finite detuning from the resonance, which can be comparable to higher-order contributions, thus 'shielding' the fragmentation of the lowest order Hamiltonian and slowing down the dynamics.

SUPPLEMENTARY NOTE 6: SCALING ANALYSIS OF THE STEADY-STATE IMBALANCE AND THE ENTANGLEMENT ENTROPY

Here, we study the system-size scaling of the long-time dynamics in a clean system without spin-dependent tilt and harmonic confinement for a large range of tilts: We choose a weak tilt $\Delta = 1J$, an intermediate tilt $\Delta = 3J$ and a large tilt $\Delta = 10J$. We focus on the dynamics close to the resonant point $|U| \approx 2\Delta$, and consider an initial Néel-ordered CDW state. This state has a symmetric charge distribution with respect to the center site and thus its dipole moment coincides with that of a homogeneous charge distribution. In Supplementary Fig. 5a we show numerical simulations of the imbalance $\mathcal{I}$ up to late times for different system sizes. In the large tilt regime, we find a stable imbalance for all system sizes, whereas the intermediate and weak tilt regime show an imbalance decay. This decay is very weak in the intermediate tilt regime and a conclusive answer on whether and at what timescale the imbalance decays to zero cannot be given. In contrast, the imbalance calculated with the effective Hamiltonian $\hat{H}^{\text{res}}_{\text{eff}}$ [Supplementary Eq.(19), Supplementary Eq. (14)] is stable (grey shaded trace in Supplementary Fig. 5a), as expected due to the absence of higher-order terms in the perturbative construction. Additionally, we find that the imbalance weakly scales down with system size. For small tilts, we clearly observe a decay of the imbalance to zero for large enough system sizes.

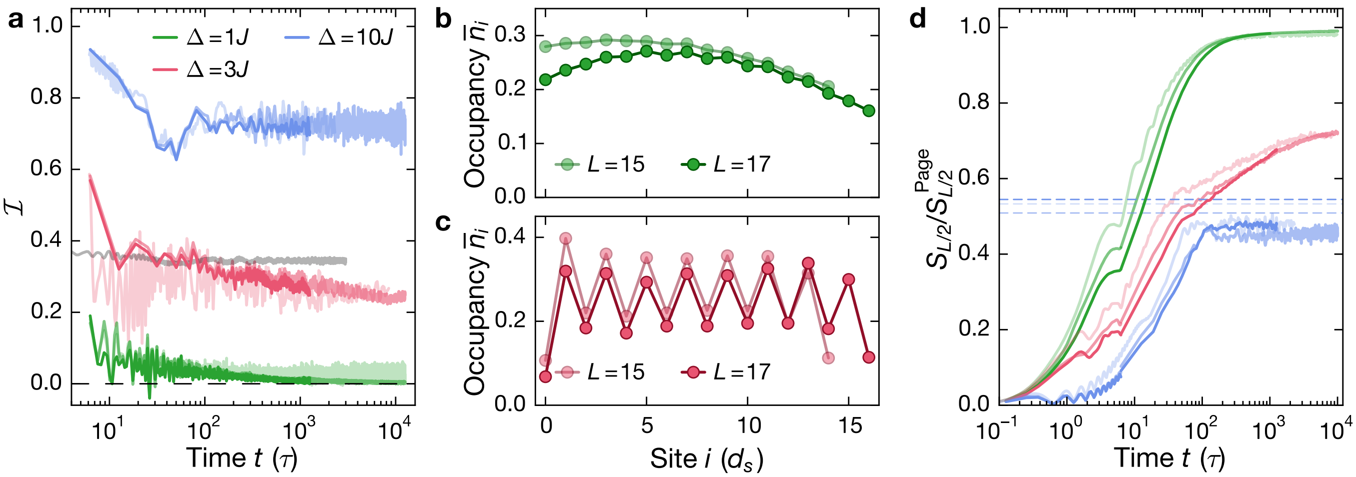

Supplementary Figure 5. **Finite-size scaling analysis of imbalance, entanglement entropy and occupancy.** **a** Long-time behavior of imbalance $\mathcal{I}$ for system sizes $L = 13, 15, 17$ and $(\Delta = 10J, U = 19.85J)$ (blue), $(\Delta = 3J, U = 4.75J)$ (red) and system sizes $L = 12, 14, 16$ for $(\Delta = 1J, U = 4.75J)$ (green). The grey line corresponds to a simulation of the imbalance according to the effective Hamiltonian $\hat{H}_{\text{eff}}^{\text{res}}$ [Supplementary Eq.(19), Supplementary Eq. (14)] for $L = 15$ and $\Delta = 3J$ up to 3000τ. Fluctuations in the data are reduced by using a running average with a time-window of 10τ. **b** ,**c** Time-averaged on-site occupancy $\overline{n}_i = 10/T \int_{0.9T}^{T} n_i dt$ for system sizes $L = 15, 17$ and **b** $(\Delta = 1J, U = 4.75J)$ and **c** $(\Delta = 3J, U = 4.75J)$. The time average was performed with $T = 12600\tau$ for $L = 15$ and $T = 1260\tau$ for $L = 17$. **d** Long-time behavior of the half-chain entanglement entropy $S_{L/2}$ normalized to the Page value $S_{L/2}^{\text{Page}}$ within the $(N_\uparrow, N_\downarrow)$ symmetry sector for the same parameters as in (a) and system sizes $L = 13, 15, 17$. The dashed horizontal lines shows the entanglement entropy of a random state within the fragment $\mathcal{K}^{\text{res}}$ containing the Néel-ordered CDW initial state. Increasing opacity corresponds to increasing system size. All calculations were done using ED.

Note that, while we used $L = 13, 15, 17$ for the intermediate and large tilt regime to minimize edge effects with an unoccupied odd site at the left and the right end of the system, we choose $L = 12, 14, 16$ for the weak tilt regime. In this regime, the initial CDW relaxes to a potentially thermal density distribution and such a distribution only has zero imbalance for an equal number of even and odd sites. Additionally, the breathing amplitude of the dynamics for $\Delta = 1J$ is four sites and boundary effects cannot be easily prevented by including an empty site at the edges. We confirm in Supplementary Fig. 5b that the on-site occupancy shows no more memory of the initial CDW order in the regime of weak tilt, consistent with a zero imbalance. For the intermediate tilt in Supplementary Fig. 5c, we clearly find a remaining CDW order.

In Supplementary Fig. 5d we show numerical simulations of the half-chain entanglement entropy $S_{L/2}$, normalized to the Page value $S_{L/2}^{\text{Page}}$ [31–33]. The Page value $S_{L/2}^{\text{Page}}$ is the half-chain entanglement entropy of a pure random state within the symmetry sector fixed by particle number $N_\uparrow, N_\downarrow$. The half-chain entanglement entropy of an ergodic system at infinite temperature is in general expected to reach $S_{L/2} = S_{L/2}^{\text{Page}}$. In the weak tilt regime, the half-chain entanglement entropy converges towards the thermal Page value for large enough system sizes, which is consistent with a lack of memory of the initial state as observed with the imbalance and the on-site occupancy. For an intermediate tilt, we observe a sub-thermal entanglement entropy, growing only very slowly at late times, which is consistent with the finite imbalance up to the latest times accessible in the simulations. For large tilt, the entanglement entropy reaches a plateau, which slightly depends on the system size. This saturation value of the entanglement entropy is slightly smaller then the entanglement entropy of a random state within the fragment $\mathcal{K}^{\text{res}}$ (blue dashed lines for the different system sizes) in which the initial state is contained.

SUPPLEMENTARY NOTE 7: EXPERIMENTAL SEQUENCE

General description

We create a degenerate Fermi gas of ^{40}K atoms in a crossed beam dipole trap. While the non-interacting traces are measured with a spin-polarized gas with all atoms in the state $|\downarrow\rangle = |F = 9/2, m_F = -9/2\rangle$, we work with an equal mixture of the states $|\uparrow\rangle = |F = 9/2, m_F = -7/2\rangle$ and $|\downarrow\rangle$ when studying interacting dynamics. Details of both the cooling sequence and the preparation of the spin-polarized gas can be found in a previous publication [34]. The initial state preparation starts with repulsively loading the atoms at a scattering length of $a = 100a_0$ into a three-dimensional (3D) optical lattice by a series of linear ramps (see Supplementary Fig. 7). The scattering length can be tuned

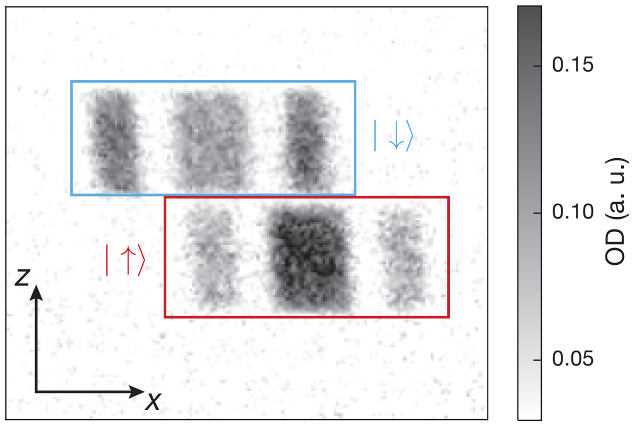

Supplementary Figure 6. **Exemplary raw image.** The two spin states $|\uparrow\rangle$, $|\downarrow\rangle$ are spatially separated using Stern-Gerlach resolved time-of-flight imaging to extract the imbalances of each component $\mathcal{I}^\sigma$ independently. The populations of first and third band are extracted via a pixel sum of the optical density (OD).

with a Feshbach resonance between the two states of the spin mixture centered at 202.1 G. While the repulsive scattering length suppresses the formation of doubly-occupied sites (doublons) during the loading, we extinguish any residual doublons by applying a 100 µs off-resonant light pulse right after loading the deep lattice [35]. The off-resonant light pulse results in light assisted collisions, which remove doublons without harming atoms on singly-occupied sites (singlons). Afterwards, we end up with singlons in an array of 1D tubes to which the dynamics is restricted on the observed timescales due to the deep orthogonal lattices. Using Gaussian fits to the atom cloud in the lattice, we characterize the 4σ width of the central tubes to $L_{\mathrm{exp}} = 290$ sites. Along the y direction and the z direction, we populate about 150 sites and 22 sites, respectively.

We create the tilted lattice by applying a magnetic field gradient with a single coil. This coil, however, not only creates a field gradient, but additionally a strong homogeneous magnetic field component up to 110 G is present, which adds to the homogeneous Feshbach field created by a pair of Helmholtz coils. Independent control of both the tilt and the interaction strength requires a tilt-dependent reduction of the Feshbach field and leads to extended wait times to reach stable currents through the coils. We use a wait time of 140 ms before time evolution and another 136 ms after time evolution before the band transfer to ensure a stable Feshbach field (see Supplementary Fig. 7). Residual dynamics within a 1D tube during the wait time are suppressed by holding the atoms in strongly tilted double wells. All experiments throughout this work employ Stern-Gerlach resolved absorption imaging after 6.4 ms time-of-flight (Supplementary Fig. 6).

CDW preparation and spin-resolved imbalance readout

After creating an array of 1D tubes in a deep 3D optical lattice, we ramp up the short lattice $\lambda_s = 532\,\mathrm{nm}$ in addition to the long lattice $\lambda_l = 1064\,\mathrm{nm}$ along the x direction at a superlattice phase of $\phi = 0.44\pi$ within 200 µs. Here, we use the convention that a symmetric double-well potential is realized for $\phi = k \cdot \pi$, with $k = \mathbb{Z}$. This creates strongly tilted double wells with one atom located on the low-energy site of each double well (even site), while the high energy site (odd site) is empty. This charge-density wave (CDW) state is time evolved in all experiments throughout this work. After time evolution, we apply a band transfer technique in the superlattice [36, 37], which maps atoms on odd sites (high-energy site of each double well) into the third band of the long lattice, while atoms on even sites remain in the first band. Here, we require a different superlattice phase of $\phi = 0.15\pi$. Afterwards we perform bandmapping and Stern-Gerlach resolved absorption imaging to evaluate the spin-resolved imbalance. The Stern-Gerlach gradient and the magnetic field gradient during time evolution are created by the same coil.

A large enough spatial separation of the two spin states $|\uparrow\rangle$ and $|\downarrow\rangle$ during Stern-Gerlach resolved bandmapping is achieved by applying a Landau-Zener sweep before the band transfer to convert atoms from $|\uparrow\rangle = |F = 9/2, m_F = -7/2\rangle$ to $|\rightarrow\rangle = |F = 9/2, m_F = -5/2\rangle$. This sweep is performed at a set magnetic field of 231.6 G, corresponding to the zero crossing of the Feshbach resonance between the two states $|\downarrow\rangle$ and $|\rightarrow\rangle$, centered around 224.2 G. We perform a linear frequency ramp with a duration of 10 ms centered at 51.87 MHz with a deviation of 1 MHz. Switching off interactions between these two states ensures the absence of interband oscillations after the transfer to the third band. Additionally, non-interacting bandmapping results in sharper edges of the absorption images and improves the accuracy of the imbalance measurement. A sample raw image used for data acquisition is shown in Supplementary Fig. 6.

Measuring a perfect imbalance equal to one can be compromised by many artifacts such as an imperfect initial state preparation and a finite transfer efficiency of the population on odd sites into the third band. In order to calibrate these imperfections, we take two different sets of images. The first set measures the highest possible initial imbalance (around 0.92(2)) with no evolution time. The second set measures the imbalance after 25 ms evolution time without tilt, which is supposed to yield zero. We then calculate a matrix that maps the measured imbalances for these two sets to 1 (first set) and 0 (second set). In particular, we have to determine a 2×2-matrix A^σ, for each state $\sigma = \uparrow, \downarrow$, which satisfies

$$\begin{pmatrix} n^\sigma_{e,1} & n^\sigma_{e,2} \\ n^\sigma_{o,1} & n^\sigma_{o,2} \end{pmatrix} A^\sigma = \begin{pmatrix} 1 & 0.5 \\ 0 & 0.5 \end{pmatrix} . \tag{22}$$

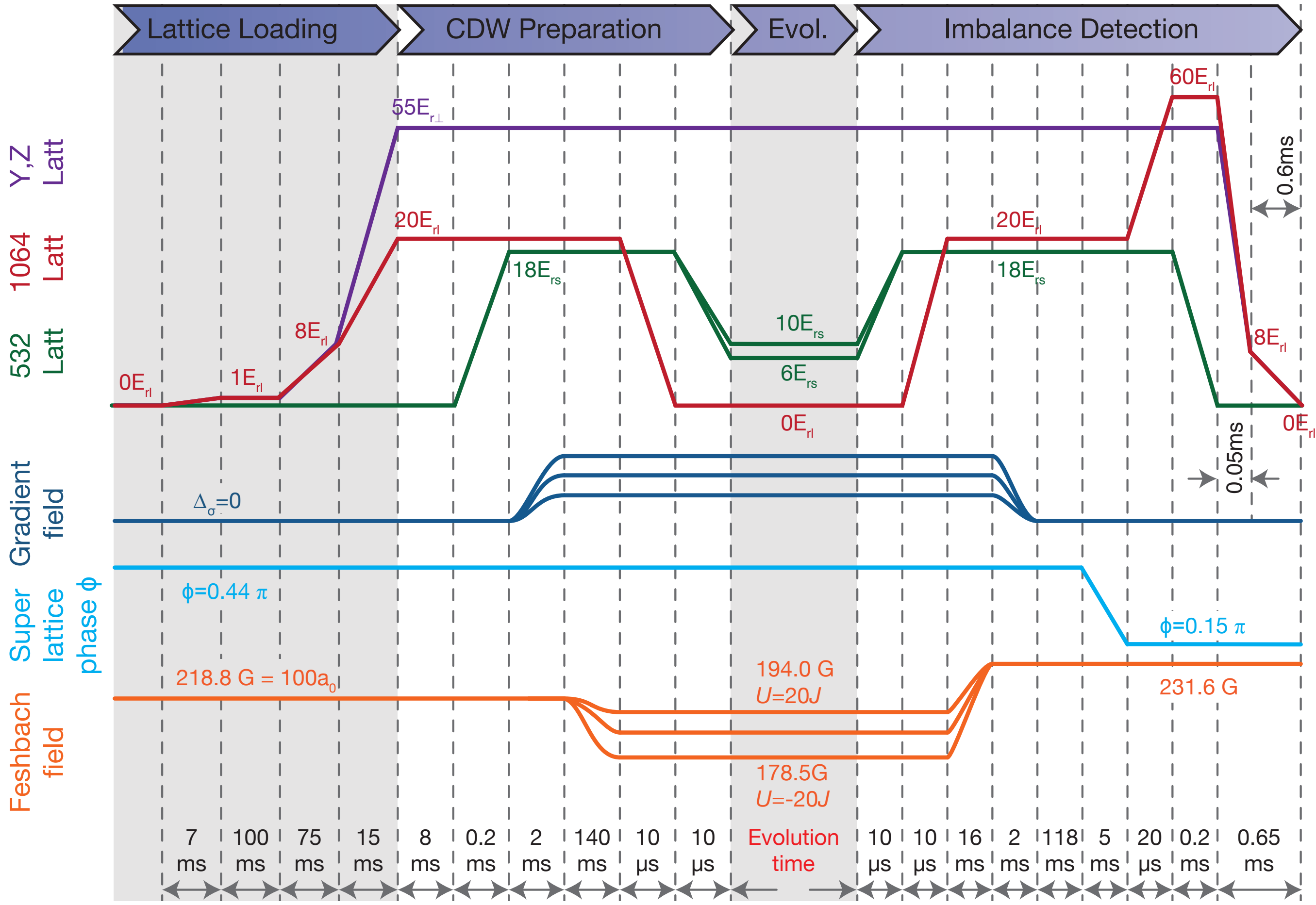

Supplementary Figure 7. **Timing protocol of the experimental sequence.** Schematic showing the lattice depths, the superlattice phase, Feshbach field ramps and the gradient field ramps for loading, CDW preparation, time evolution and detection of the imbalance.

Here, $n^{\sigma}_{e,i}$ ($n^{\sigma}_{o,i}$) denote the relative atom number on even (odd) sites for the respective spin state and $i = 1, 2$ refers to the imbalance in the respective set (first or second set). This matrix is then used to rescale the measured imbalance for each spin component.

SUPPLEMENTARY NOTE 8: CREATING A HOMOGENEOUS POTENTIAL

Before loading into the lattice the atoms are confined by three dipole trap beams. The horizontal beams along x (dynamics) and y are elliptical ($30 \times 300\,\mu$m) and the vertical beam along the z-direction is circular with a Gaussian beam waist of $150\,\mu$m. All optical lattice beams have the same size as the z-dipole trap and are blue-detuned, thus providing an anti-trapping potential. A flat potential during the Bloch oscillations can be achieved by compensating the confinement of the vertical dipole trap with the anti-confinement of the optical lattices. The horizontal traps should only marginally contribute to the total confinement and we find the optimal configuration if the x-dipole trap is switched off and the y-dipole trap provides a very weak confinement during the time evolution. The confinement is optimized by fixing the time to four Bloch cycles ($t = 4T_{\downarrow}$) and scanning the dipole trap strength on maximal imbalance. This method works because the confinement does not lead to a frequency change of the oscillations.

SUPPLEMENTARY NOTE 9: CREATING A LINEAR POTENTIAL

The tilt is created by applying a magnetic field gradient. The energy E^{σ} of each state $|\sigma\rangle$ in the $F = 9/2$ hyperfine ground-state manifold in the presence of a magnetic field can be analytically calculated using the Breit-Rabi formula and a magnetic field gradient results in a linear potential $\Delta_{\sigma} i$ according to $\Delta_{\sigma} = \frac{dE^{\sigma}}{dB}\partial_x B$. The first factor $\frac{dE^{\sigma}}{dB}$ causes the tilt to be spin-dependent and in the Zeeman limit of weak field $B \to 0$, we get $\Delta_{\uparrow}/\Delta_{\downarrow} = 7/9 = 78\%$. With increasing field, the spin-dependence reduces (in the Paschen-Back limit $B \to \infty$

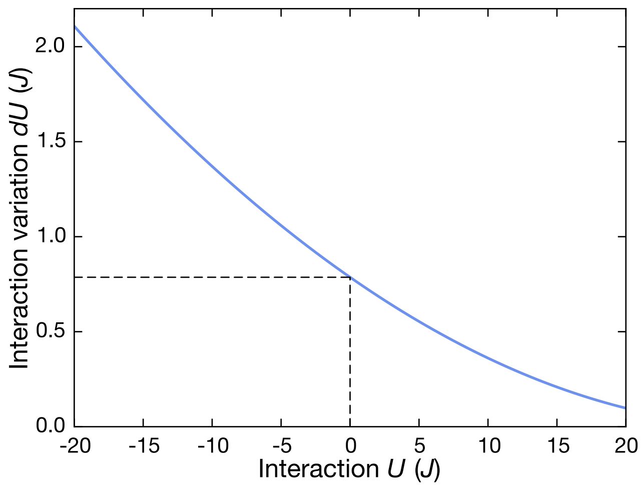

Supplementary Figure 8. **Impact of interaction averaging.** Variation of the interaction strength across one tube with 290 lattice sites, a tilt of $\Delta_\downarrow/h = 1.8\,\mathrm{kHz}$ and tunneling rate $J/h = 540\,\mathrm{Hz}$. For the orthogonal lattices we use $55E_r$.

we have $\Delta_\uparrow/\Delta_\downarrow = 1$) and for the magnetic field used in this work ($B \approx 210\,\mathrm{G}$), we have $\Delta_\uparrow/\Delta_\downarrow = 90.6\%$. The second factor $\partial_x B$ is spin-independent and describes the magnetic field gradient, which we create with a single coil close to the atom cloud. The magnetic field along the 1D tubes is given by $B(x) = B_0 + a(x - x_0) + b(x - x_0)^2$ plus higher orders which are negligible for our parameters. Here, x_0 is the center of the coil, x is the relative distance of the atomic cloud, a is the field gradient and b is the field curvature. The coil has a diameter of 25 mm, 20 windings and a mean distance to the atoms of 26.5 mm. Currents up to 55 A are applied. We note that the magnetic field generated by this configuration mainly possesses a large homogeneous contribution and a gradient part producing the linear potential. The weak field curvature part adds to the harmonic confinement of the lattice and dipole beams.

SUPPLEMENTARY NOTE 10: INTERACTION AVERAGING

The magnetic field gradient used for generating the tilt causes a local variation of the total magnetic field. Since we use the total magnetic field to set the interaction strength with a Feshbach resonance, the variation of the total magnetic field also induces a variation of the interaction strength over the length of a tube. From the typical center tube length of 290 lattice sites (4σ width of the cloud) and the width of the Feshbach resonance we can calculate the impact of this averaging effect for a certain tilt and lattice configuration. Due to the Gaussian density distribution of the cloud, assuming 290 sites as tube length overestimates the averaging effect and gives a crude upper bound. Supplementary Fig. 8 shows the strength of the averaging effect $U_{\mathrm{var}} = U \pm dU$ as a function of the central interaction strength for a 1D system with $8E_r$ primary lattice depth. For shallower lattices this effect diminishes. Note that while the Stark model exhibits a dynamical U vs. $-U$ symmetry the interaction averaging slightly breaks this symmetry. It also underlines why the non-interacting data we show in this work is mostly taken with a spin-polarized sample. Even if the scattering length is set to zero via the Feshbach resonance, small residual interactions remain due to the averaging.

SUPPLEMENTARY NOTE 11: CALIBRATION OF PARAMETERS

Curvature α and trap frequency ω_h

Any non-linear correction to the linear on-site potential leads to a spectrum of Bloch oscillation frequencies in the system that are averaged over in the measurement. The non-linearity in our system is caused by the residual harmonic confinement, modelled as a quadratic correction term to the linear potential, $\Delta_\downarrow i + \alpha(i - i_0)^2$, where i_0 is the center of the lattice. The observed Bloch oscillation is then a sum of Bloch oscillations with frequencies ranging between $\Delta_\downarrow - 2\alpha L/2$ and $\Delta_\downarrow + 2\alpha L/2$ with a step of 2α and a system size of L sites. In order to understand the result of such a sum, consider, for instance, a sum of sinusoidal oscillations,

$$\begin{aligned} f(t) &= \sum_{i=-L/2}^{L/2} \cos\bigl(2\pi(\Delta_\downarrow + \alpha i)t\bigr) \\ &= \cos\bigl(2\pi\Delta_\downarrow t\bigr)\frac{\sin\bigl(2\pi(L+1)\alpha t\bigr)}{\sin(2\pi\alpha t)}. \end{aligned} \tag{23}$$

This is an oscillation at frequency $\Delta_\downarrow$ together with a beat note envelope at a frequency $(L+1)\alpha \approx L\alpha$ and nodes at $1/(2L\alpha)$. The Bessel-type Bloch oscillations, which can be expressed as sum of few sinusoidal oscillations, would behave in a qualitatively similar manner. Therefore, we expect a collapse at time $T_c \approx 1/(2L\alpha)$, before the imbalance revives. We use numerical calculations of the imbalance time trace for a non-interacting system in a lattice of size $L = 290(20)d$ to determine the value of α, as a fit parameter. Corresponding to an experimentally measured imbalance time trace $\mathcal{I}^\downarrow(t_j) : j = 1, 2, \cdots, n$, where n is the number of data points in time, we compute, numerically, the trace $\mathcal{I}^\downarrow_{\mathrm{num}}(t_j; J, \Delta_\downarrow, \alpha)$ and then minimize $\sum_j |\mathcal{I}^\downarrow(t_j) - \mathcal{I}^\downarrow_{\mathrm{num}}(t_j; J, \Delta_\downarrow, \alpha)|^2$ over alpha to determine the fit value. The harmonic confinement is extracted in Supplementary Fig. 9. We find a collapse time of $T_c = 8\,\mathrm{ms}$, corresponding to $\alpha = h \cdot 0.216\,\mathrm{Hz}$ and $\omega_h/2\pi = \sqrt{\frac{\alpha\hbar}{md^2\pi}} = 39\,\mathrm{Hz}$. Due to the local nature of the dynamics in the Stark Hamiltonian, α is the important energy scale for the dynamics, characterizing the amount of curvature, experienced by every single atom. In our

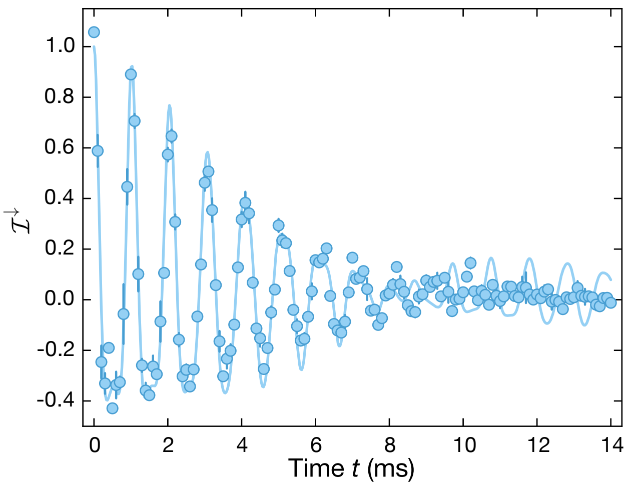

Supplementary Figure 9. **Calibration of the harmonic confinement.** Imbalance $\mathcal{I}^{\downarrow}$ for a spin-polarized gas at $\Delta_{\downarrow} = 1.8J$ and $J = h \cdot 540\,\mathrm{Hz}$. Each data point is averaged twice and error bars denote the SEM. The solid line is a fit to the data using an ED calculation, which includes the harmonic confinement. The resulting collapse time is $T_c = 8\,\mathrm{ms}$.

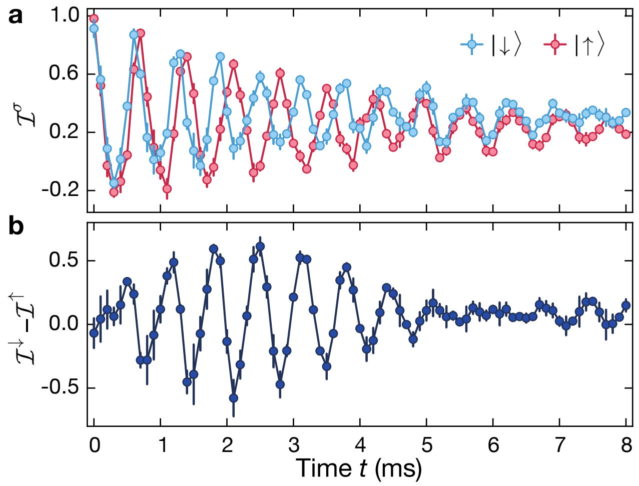

Supplementary Figure 10. **Spin-resolved Bloch oscillations. a** Typical calibration measurement of the tilt Δ_σ for both spin-components using the spin-resolved imbalance $\mathcal{I}^{\sigma}$. Here, $\Delta_{\downarrow}/h = 1.60(1)\,\mathrm{kHz}$ and we extract a frequency difference of $(\Delta_{\downarrow} - \Delta_{\uparrow})/h = 170(2)\,\mathrm{Hz}$, which is in reasonable agreement with the calculated difference. Each data point is averaged four times and error bars denote the SEM. **b** Imbalance difference between $|\downarrow\rangle$ and $|\uparrow\rangle$. The resulting pattern exhibits a beat note similar to the trigonometric identity $\cos(\omega_1 t) - \cos(\omega_2 t) = -2\sin\big((\omega_1+\omega_2)t/2\big)\sin\big((\omega_1-\omega_2)t/2\big)$.

system, the tilt is on the order of $\Delta_{\downarrow} \approx h \cdot 1000\,\mathrm{Hz}$ and the curvature is very weak ($\alpha/\Delta_{\downarrow} \approx 10^{-4}$). Theoretically, the imbalance oscillations should revive partially, but due to anharmonic confinement, residual onsite disorder and other dephasing mechanisms we cannot see such revivals. All these artifacts can affect the envelope of the Bloch oscillations in addition to the dephasing of the harmonic confinement and are also included in the extracted collapse time. Hence, extracting the harmonic confinement from the collapse time yields an upper bound for the true harmonic confinement.

Spin-dependent tilt Δ_σ

The setup consists of one pair of coils in Helmholtz configuration to generate a homogeneous magnetic field B_z along the vertical z direction for controlling the interactions between the two spin states by a Feshbach resonance. Additionally, a gradient coil is used to create a magnetic field B_x consisting of a homogeneous field B_{x0} and the field gradient $\frac{\mathrm{d}B_x}{\mathrm{d}x}$ along the x direction. Therefore, the total field is $B_0 = \sqrt{B_x^2 + B_z^2}$. Expanding the above expression up to first order, we get for the total field

$$
\begin{aligned}
B_0(x) &= \sqrt{B_z^2 + B_x^2} = \sqrt{B_z^2 + \left(B_{x0} + x\frac{\mathrm{d}B_x}{\mathrm{d}x}\right)^2} \\
&\simeq B_z + \frac{B_{x0}^2}{2B_z} + \frac{B_{x0}}{B_z}\cdot x\frac{\mathrm{d}B_x}{\mathrm{d}x}.
\end{aligned} \tag{24}
$$

In the last step we used that B_z is the strongest contribution such that the square root can be expanded up to first order and we neglected the term of the squared gradient. We note that the strength of the gradient is reduced by the vertical field component and amplified by the homogeneous horizontal field. It follows that the calibration of tilt and interactions has to be an iterative process, since these quantities strongly depend on each other. We determine the required vertical magnetic field B_z in the presence of a current I_G in the gradient coil in order to generate a fixed total homogeneous magnetic field B_0. For this sake we employ an RF sweep from $|\downarrow\rangle$ to $|\uparrow\rangle$, whose frequency is set to the value corresponding to B_0. We find the relation

$$
B_z(I_G) = B_0 - \frac{(aI_G)^2}{B_0} + bI_G\,, \tag{25}
$$

with fit parameters a and b. From Supplementary Eq. (24) it follows that $\Delta_\sigma \propto I_G^2$ where the proportionality constant depends on B_z. The current required to generate a certain tilt Δ_σ can thus be expressed as

$$
I_G = c\sqrt{\Delta_\sigma \cdot B_z} \tag{26}
$$

with constant c. We calibrate this fit parameter using single-particle Bloch oscillations and extract the oscillation frequency, set by the tilt Δ_σ, with the analytical model using the first four oscillations to minimize effects of the damping. A typical calibration measurement is illustrated in Supplementary Fig. 10 for a spin-mixture

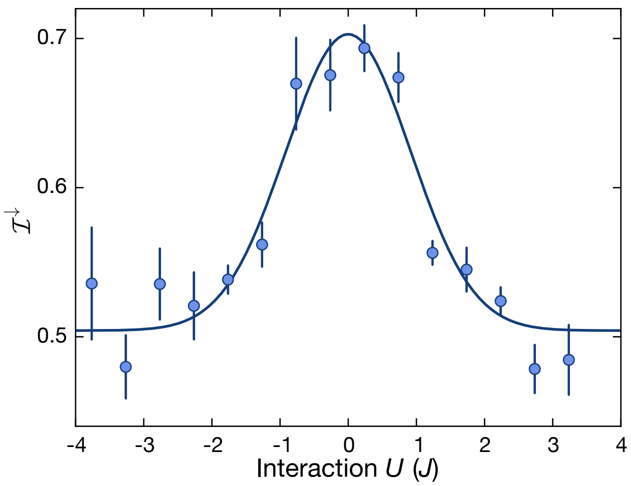

Supplementary Figure 11. **Calibration of the zero-crossing.** Imbalance of one spin-component $\mathcal{I}^{\downarrow}$ versus interaction strength. We use a tilt $\Delta_{\downarrow}/h = 1.2\,\mathrm{kHz}$ and measure the imbalance after $t = h/\Delta_{\downarrow}$. The tunneling rate is $J/h = 540\,\mathrm{Hz}$. The solid line is a Gaussian fit to capture the peak of the imbalance, corresponding to the zero-crossing of the Feshbach resonance. Each data point consists of four independent measurements and error bars denote the SEM.

at $\Delta_{\downarrow} = h \cdot 1.60(1)\,\mathrm{kHz}$. We clearly see the different tilts in the oscillation frequency of the respective spin component. Finally, from Supplementary Eq. (24) and Supplementary Eq. (26) we see that it requires an iterative adaption of the current and the vertical field, since they are strongly correlated. In the experiment we do two full iteration steps until the values sufficiently converge.

Lattice depth

All optical lattices are calibrated using Kapitza-Dirac scattering with a Bose-Einstein condensate of ^{87}Rb and the lattice depth calibration is then converted to ^{40}K. While this technique in principle also calibrates the tunneling J in Eq. (1) in the main text, we determine the tunneling J for the data in Fig. 2 and Fig. 3 in the main text directly by using a fit of Eq. (2) to the short-time dynamics ($U = 0J$, spin-polarized). We only use times $t \leq 1.5\,\mathrm{ms}$ such that the damping of the oscillations is negligible (the collapse time is $T_c = 8\,\mathrm{ms}$). For a set lattice depth of $8E_{rs}$ ($6E_{rs}$) the fit yields $J = h\cdot 0.54(1)\,\mathrm{kHz}$ ($J = h \cdot 0.88(2)\,\mathrm{kHz}$) and agrees in both cases well with the calculated tunnelling rate $J_{8E_{rs}} = h \cdot 0.543\,\mathrm{kHz}$ and $J_{6E_{rs}} = h \cdot 0.896\,\mathrm{kHz}$. Note that the solid line in Fig. 3b in the main text is a plot of Eq. (3), where we use $J = h\cdot 0.54(1)\,\mathrm{kHz}$, obtained from the short time dynamics, without any additional free parameter. The excellent agreement of analytic prediction and data at late times emphasizes the accuracy of calibrating J with the short time dynamics.

Onsite interaction U

The non-interacting point of the Feshbach resonance between the states $|{\uparrow}\rangle$ and $|{\downarrow}\rangle$ is calibrated with Bloch oscillations by taking advantage of the interaction-induced damping and the dynamical symmetry between repulsive and attractive interactions. For every tilt $\Delta_{\downarrow}$ we fix the time t at $t = T_{\downarrow} = h/\Delta_{\downarrow}$, while scanning the Feshbach field. In Supplementary Fig. 11 we show a typical calibration measurement, where the zero-crossing of the Feshbach resonance is well detectable as the interaction strength, which has the largest imbalance. A finite interaction U causes a strong damping, which decreases the imbalance. Since the magnetic field $B_0 = 202.1\,\mathrm{G}$ of the center of the Feshbach resonance is well known [38], the zero crossing is set by the width w_{202} plus the magnetic field of the center B_0. We use the calibration of the zero crossing to determine a precise value for the width of the Feshbach resonance: $w_{202} = 7.1(1)\,\mathrm{G}$, in agreement with the literature [15]. The same measurement was performed for the Feshbach resonance between $|{\downarrow}\rangle$ and $|{\rightarrow}\rangle$ centered at $B = 224.2\,\mathrm{G}$ [39], where we extract a width $w_{224} = 7.4(1)\,\mathrm{G}$ in agreement with the literature [40]. The characterization of the Feshbach resonance together with the calibration of the lattice depth yields a calibration for the onsite interaction U.

SUPPLEMENTARY NOTE 12: EXTRACTION OF THE INTERACTION ENERGY U_{res}

In Fig. 3e in the main text we discuss the resonant process connecting two singlons on even sites with a doublon via a second-order hopping process, with $U \simeq 2\Delta_{\downarrow}$. In Supplementary Fig. 12, we show how the interaction energy U_{res} of the resonance for different tilts $\Delta_{\downarrow}$ was extracted using a Gaussian fit $f(x) = Ae^{-(x-x_c)^2/(2\sigma^2)} + C$ to locate the minimum of the imbalance $\mathcal{I}^{\downarrow}$. Note that the naive expectation for the interaction energy $U_{\mathrm{res}} = 2\Delta_{\downarrow}$ does not apply here, because the resonance is of second order and therefore the interaction energy is renormalized such that we expect $U_{\mathrm{res}} + 8J^2/(3\Delta_{\downarrow}) = 2\Delta_{\downarrow}$ up to second-order perturbation theory.

SUPPLEMENTARY NOTE 13: DETAILS ON NUMERICAL METHODS

We use an exact diagonalization technique to simulate the long-time dynamics. The dimension of the subspace of N_σ spin σ atoms on L lattice sites is $d_\sigma = \binom{L}{N_\sigma}$. The state ψ is a $d_\uparrow d_\downarrow$ dimensional vector. The total dimension of the Hilbert space is $d_\uparrow d_\downarrow$ — H is a $d_\uparrow d_\downarrow \times d_\uparrow d_\downarrow$ matrix. For $L = 12, N_\sigma = 3$, this dimension is $220^2 = 48400$; for $L = 16, N_\sigma = 4$, it is $1820^2 = 3312400$ and for $L = 20, N_\sigma = 5$, it is $15504^2 = 240374016$. At $L = 12$, the Hamiltonian already consists of 48400^2 floating point numbers, occupying up to 75 GB of RAM. We therefore

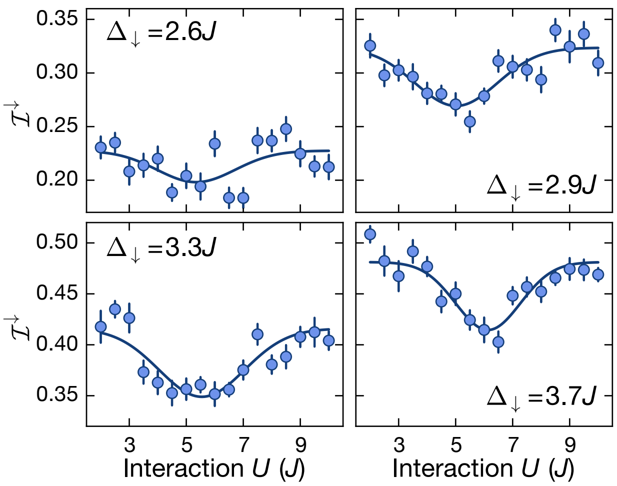

Supplementary Figure 12. **Probing the interaction energy U_{res}.** Interaction scan of the imbalance $\mathcal{I}^\downarrow$ of one spin component for different tilts $\Delta_\downarrow$. The solid line is a Gaussian fit $\mathcal{I}^\downarrow(U) = Ae^{-(U-U_{\mathrm{res}})^2/(2\sigma^2)} + C$ to the data extracting the interaction energy U_{res} for which the imbalance is minimal. The fit yields $U_{\mathrm{res}} = 5.3(6)J$ ($\Delta_\downarrow = 2.6J$), $U_{\mathrm{res}} = 5.0(2)J$ ($\Delta_\downarrow = 2.9J$), $U_{\mathrm{res}} = 5.5(2)J$ ($\Delta_\downarrow = 3.3J$) and $U_{\mathrm{res}} = 6.1(2)J$ ($\Delta_\downarrow = 3.7J$). Each data point is averaged three times over ten equally spaced times in a window between 170τ and 200τ. Error bars denote the SEM.

use the following method for the computation, which enables us to go up to $L = 20$ before using sparse matrices or a Lanczos algorithm. Below, we describe the construction of the basis, the Hamiltonian and time evolution.

Basis construction

Accounting for atom number conservation in both the spins, as mentioned before, we are working in a subspace of dimension $d_\uparrow \times d_\downarrow$. This is a system of $N_\uparrow + N_\downarrow$ fermions with a total of $2L$ fermionic modes represented by the creation operators $\hat{c}^\dagger_{1,\uparrow}, \hat{c}^\dagger_{2,\uparrow}, \cdots, \hat{c}^\dagger_{L,\uparrow}, \hat{c}^\dagger_{1,\downarrow}, \hat{c}^\dagger_{2,\downarrow}, \cdots, \hat{c}^\dagger_{L,\downarrow}$. A typical number state can be written as $\hat{c}^\dagger_{i_1,\uparrow}\hat{c}^\dagger_{i_2,\uparrow}\cdots\hat{c}^\dagger_{i_{N_\uparrow},\uparrow}\hat{c}^\dagger_{j_1,\downarrow}\hat{c}^\dagger_{j_2,\downarrow}\cdots\hat{c}^\dagger_{j_{N_\downarrow},\downarrow}|0\rangle$, where $\{i_1, \cdots, i_{N_\uparrow}\}$ and $\{j_1, \cdots, j_{N_\downarrow}\}$ are subsets (not necessarily disjoint) of $\{1, 2, \cdots, L\}$. We construct a canonical representation of this state by ordering the operators such that $i_1 < i_2 < \cdots < i_{N_\uparrow}$ and $j_1 < j_2 < \cdots < j_{N_\downarrow}$. This state can be represented by the pair of tuples $((i_1, i_2, \cdots, i_{N_\uparrow}), (j_1, j_2, \cdots, j_{N_\downarrow}))$. Next we order the tuples $\{(i_1, i_2, \cdots, i_{N_\uparrow})\}$ lexicographically to construct a list of tuples $\mathcal{V}_\uparrow$ and $\mathcal{V}_\downarrow$. The full basis would then be $\mathcal{V}_\uparrow \times \mathcal{V}_\downarrow$. In this basis, the non-interacting part of the Hamiltonian remains separable and we make use of this property to optimize the time and memory consumption.

a. Off-diagonal elements of the Hamiltonian: Note that a typical hopping term in the Hamiltonian corresponding to spin $\uparrow$ atoms not only leaves the spin $\downarrow$ part of a basis element unchanged, but also maintains the sign of the state with the trivial exception of the boundary hopping (e.g., $\hat{c}^\dagger_{1,\uparrow}\hat{c}_{L,\uparrow}$). The hopping matrix can, therefore, be written as $\hat{H}^{\mathrm{hop}}_\uparrow \otimes 1 + 1 \otimes \hat{H}^{\mathrm{hop}}_\downarrow$ where $\hat{H}^{\mathrm{hop}}_\sigma = \sum_i \hat{c}^\dagger_{i,\sigma}\hat{c}_{i+1,\sigma} + \mathrm{h.c.}$ is the $d_\sigma \times d_\sigma$ matrix corresponding to the hopping of spin σ atoms, acting on span($\mathcal{V}_\sigma$). We construct $\hat{H}^{\mathrm{hop}}_\uparrow$ and $\hat{H}^{\mathrm{hop}}_\downarrow$ separately. These two matrices are *small*, in the sense that their dimensions are $d_\uparrow$ and $d_\downarrow$, much smaller than the full Hilbert space $d_\uparrow \times d_\downarrow$. These matrices can be stored in as dense matrices even when $L = 20$ and $N_\sigma = 5$.

b. Diagonal elements: We represent the potential of a spin σ atom at site i by $V_{i,\sigma}$. We store *only* the diagonal entries of the Hamiltonian in a matrix V of size $d_\uparrow \times d_\downarrow$. The matrix element $V_{\alpha\beta}$ is the energy of the basis element corresponding to α-th tuple in $\mathcal{V}_\uparrow$ and β-th tuple in $\mathcal{V}_\downarrow$. If this basis element is $((i_1, i_2, \cdots, i_{N_\uparrow}), (j_1, j_2, \cdots, j_{N_\downarrow}))$, the energy is

$$V_{\alpha\beta} = \sum_{k=1}^{N_\uparrow} V_{i_k,\uparrow} + \sum_{k=1}^{N_\downarrow} V_{j_k,\downarrow} + U|\{i_1, \cdots, i_{N_\uparrow}\} \cap \{j_1, \cdots, j_{N_\downarrow}\}|$$

Here, $|\{i_1, \cdots, i_{N_\uparrow}\} \cap \{j_1, \cdots, j_{N_\downarrow}\}|$ is the number of elements in the intersection of $\{i_1, \cdots, i_{N_\uparrow}\}$ and $\{j_1, \cdots, j_{N_\downarrow}\}$. This is the number of doublons in the state and the last term in the above equation corresponds to the Hubbard interaction.

Intermediate time evolution

We define a $d_\uparrow \times d_\downarrow$ matrix $M^{(\psi)}$, storing the state ψ, whose $\alpha\beta$-th element is $M^{(\psi)}_{\alpha\beta} = \langle \alpha, \beta | \psi \rangle$. Here, $|\alpha\beta\rangle$ is the basis element with indices (α, β) in $\mathcal{V}_\uparrow \times \mathcal{V}_\downarrow$. The rows of $M^{(\psi)}$ correspond to spin $\uparrow$ and columns correspond to spin $\downarrow$. With this setting, the state is $M^{(\psi)}$ and the Hamiltonian, represented by the triplet $\{\hat{H}^{\mathrm{hop}}_\uparrow, \hat{H}^{\mathrm{hop}}_\downarrow, \hat{V}\}$, all of which are $d_\uparrow \times d_\downarrow$, $d_\downarrow \times d_\downarrow$ or $d_\uparrow \times d_\uparrow$ matrices. Therefore it is convenient to work in this picture rather than use the full Hamiltonian which is much bigger. The Schrödinger equation in this representation is given by

$$\dot{M^{(\psi)}} = -i\hat{H}^{\mathrm{hop}}_\uparrow M^{(\psi)} - iM^{(\psi)}\hat{H}^{\mathrm{hop}}_\downarrow - i\hat{V} \circ M^{(\psi)} \quad (27)$$

Here, $\circ$ represents element-by-element multiplication, known as Hadamard product. To see that this is the correct equation of time evolution, consider the Schrödinger equation in the standard representation

$$\dot{\psi} = -i\hat{H}^{\mathrm{hop}}_\uparrow \otimes 1\psi - i1 \otimes \hat{H}^{\mathrm{hop}}_\downarrow\psi - i\hat{H}^{diag}\psi \quad (28)$$

Here, $\hat{H}^{\mathrm{diag}}$ is a $d_\uparrow d_\downarrow \times d_\uparrow d_\downarrow$ diagonal matrix consisting of the elements in $\hat{V}$. The first term in the above equation reads $\hat{H}^{\mathrm{hop}}_\uparrow \otimes 1\psi = \sum_{\alpha,\beta}\sum_\gamma \hat{H}^{\mathrm{hop}}_{\uparrow\alpha\gamma} M^{(\psi)}_{\gamma\beta}|\alpha\beta\rangle =$

$\sum_{\alpha,\beta}(\hat{H}_{\uparrow}^{\text{hop}}M^{(\psi)})_{\alpha\beta}\,|\alpha\beta\rangle$. The second terms reads $1\otimes\hat{H}_{\downarrow}^{\text{hop}}\psi = \sum_{\alpha,\beta}\sum_{\gamma}\hat{H}_{\downarrow\beta\gamma}^{\text{hop}}M_{\alpha\gamma}^{(\psi)}\,|\alpha\beta\rangle = \sum_{\alpha,\beta}(M^{(\psi)}\hat{H}_{\downarrow}^{\text{hop}})_{\alpha\beta}\,|\alpha\beta\rangle$. Thus the first term corresponds to a *left* multiplication of $M^{(\psi)}$ by $\hat{H}_{\uparrow}^{\text{hop}}$ and the second term corresponds to a *right* multiplication by $\hat{H}_{\downarrow}^{\text{hop}}$. The third term reads $\hat{H}^{\text{diag}}\psi = \sum_{\alpha,\beta}\hat{V}_{\alpha\beta}M_{\alpha\beta}^{(\psi)}\,|\alpha\beta\rangle$; this corresponds to a *term-by-term* multiplication of $M^{(\psi)}$ by $\hat{V}$. Using Trotter-Suzuki decomposition to solve Supplementary Eq. (27) yields

$$M^{(\psi)}(t+\delta t)\approx e^{-i\delta t\circ\hat{V}}\circ e^{-i\delta t\hat{H}_{\uparrow}^{\text{hop}}}M^{(\psi)}(t)e^{-i\delta t\hat{H}_{\downarrow}^{\text{hop}}} \quad (29)$$

Here, $e^{-i\delta t\circ\hat{V}}$ is an element-by-element exponentiation.

Error estimates

The key problem here is to estimate the error accumulated due to the Trotter-Suzuki decomposition. We consider n trotter steps per Bloch period, that is, $\delta t=\frac{1}{n\Delta}$. We consider time evolutions up to time T. If $\mathcal{I}_n(t)$ is the imbalance computed using n trotter steps per Bloch period, our objective is to estimate $||\mathcal{I}-\mathcal{I}_n||$ where $\mathcal{I}(t)$ is the imbalance at $n=\infty$, which we can calculate for small system sizes using Supplementary Eq. (28), by exponentiating the full Hamiltonian. We use the standard $\mathcal{L}^p$-norm, i.e., $||\mathcal{I}_n-\mathcal{I}||_p=\left(\int_0^T|\mathcal{I}_n(t)-\mathcal{I}(t)|^p dt\right)^{\frac{1}{p}}$ for $p=1,2$ and ∞. In the latter case, $||\mathcal{I}_n-\mathcal{I}||_\infty=\max(|\mathcal{I}(t)-\mathcal{I}_n(t)|)$. Intuitively, $p=1$ corresponds to the "average case" distance between $\mathcal{I}$ and $\mathcal{I}_\infty$ and $p=\infty$ represents the "worst case" distance.

We numerically show that $a_n=||\mathcal{I}-\mathcal{I}_n||=O\left(\frac{1}{n^2}\right)$. In other words, $||\mathcal{I}-\mathcal{I}_n||\to 0$ as $1/n^2$. To see this, let us consider the sequence $b_n=||\mathcal{I}_{n+r}-\mathcal{I}_n||$, for a fixed k. Supplementary Fig. 13a shows that $b_n=O\left(\frac{1}{n^3}\right)$. Moreover, from triangle inequality, $|a_{n+r}-a_n|\le b_n$ and therefore, $a_n=O\left(\text{cusum}\left(\frac{1}{n^3}\right)\right)=O\left(\frac{1}{n^2}\right)$. Here, cusum$(x)$ is the cumulative sum. Thus, for large n and some k, we can assume that $a_n\sim k^2a_{kn}$ and it follows from the triangle inequality, $a_n-a_{kn}\le||\mathcal{I}_{kn}-\mathcal{I}_n||$ that $a_n\approx\frac{k^2}{k^2-1}||\mathcal{I}_{kn}-\mathcal{I}_n||$. The RHS of last inequality can be computed numerically. Thus, we obtain

$$||\mathcal{I}_m-\mathcal{I}||\approx\frac{k^2n^2}{(k^2-1)m^2}||\mathcal{I}_{kn}-\mathcal{I}_n|| \quad (30)$$

We use $n=100$ and $k=5$ in Supplementary Fig. 13b. We use the above expression to estimate $a_n=||\mathcal{I}-\mathcal{I}_n||$, for $L=12$ and higher, and choose n such that $a_n\le 10^{-3}$.

General error analysis In the previous section, the error accumulated due to the Trotter-Suzuki approximation was analysed using the deviations in the imbalance as the figure of merit. While this approach is relevant for our purpose, the deviations in the state vector itself would be relevant in a more general context. Indeed, if $\psi(t)$ is the many body state vector at time t and $\psi_n(t)$ is the state vector computed using a Trotter-Suzuki approximation using n Trotter steps per Bloch period, the deviation $||||\psi_n(t)-\psi(t)||_2||_p$ can be related to the deviation in any arbitrary observable $\hat{O}$ (Note that we use the standard 2-norm to quantify the distance between $\psi(t)$ and $\psi_n(t)$ at a given time t and then use a $p-$norm to quantify the overall deviation). For instance, consider $\langle\psi|\hat{O}|\psi\rangle-\langle\psi_n|\hat{O}|\psi_n\rangle=\langle\psi|\hat{O}|\psi\rangle-\langle\psi|\hat{O}|\psi_n\rangle+\langle\psi|\hat{O}|\psi_n\rangle-\langle\psi_n|\hat{O}|\psi_n\rangle=\langle\psi-\psi_n|\hat{O}|\psi\rangle+\langle\psi_n|\hat{O}|\psi-\psi_n\rangle\le 2||\hat{O}||||\psi-\psi_n||_2$. Here, $||\hat{O}||$ is the operator norm, i.e., the largest singular value of $\hat{O}$, assuming $\hat{O}$ is finite-dimensional. Thus, the deviation $||\langle\psi_n(t)|\hat{O}|\psi_n(t)\rangle-\langle\psi(t)|\hat{O}|\psi(t)\rangle||_p$ can be estimated, loosely, using $||||\psi_n(t)-\psi(t)||_2||_p$. In Supplementary Fig. 14a we show that $||||\psi_n(t)-\psi(t)||_2||_p\sim\frac{1}{n}$ for a system with $L=8$ sites and $N_\sigma=2$. It is interesting to note that the imbalance converges much faster $\sim\frac{1}{n^2}$.

Long-time evolution

At the outset it appears that by making a Trotter-Suzuki decomposition, we lose the logarithmic scaling of complexity in time of the scaling-and-squaring procedure of matrix exponentiation. That is, for a long time T, the unitary $e^{-i\hat{H}T}$ can be computed by scaling T to $T/2^n$ for some integer n, computing $e^{-i\hat{H}T/2^n}\approx 1-i\hat{H}T/2^n-\hat{H}^2/2T^2/4^n$ and squaring it repeatedly, n times. The complexity of this procedure is linear in n. For a fixed tolerance, it is logarithmic in T, enabling a computation of very long-time dynamics. Although it appears that we lose this advantage while using the Trotter-Suzuki decomposition, we show below that the scaling can be improved, asymptotically.

In Supplementary Fig. 13c we show how the error of the computation increases with time for a fixed trotter step, suggesting at least a linear growth in T. Moreover, for fixed trotter step, the computational time also grows linearly in T. Thus, for a fixed tolerance, the computational time grows at least quadratically in T. We show below that this can be improved to a linear scaling in T. The idea is to reduce the error in the computation using an elimination technique so that it scales down faster. Let $T\gg 1/\Delta$ be a long time up to which we intend to compute the evolution the system. That is, we want to compute $\psi(T)$. Let us suppose that we computed $\psi(T)$ twice, using the above described procedure, once using $\delta t=1/(n\Delta)$ and the second time using $\delta t=1/((n+1)\Delta)$ and obtained two state vectors $\psi_n(T)$ and $\psi_{n+1}(T)$. From the above considerations, we know that $||\psi(T)-\psi_n(T)||=\kappa/n+O(1/n^2)$ for some κ. We consider a linear combination of $\psi_n(T)$ and $\psi_{n+1}(T)$:

$$\tilde{\psi}_{n,2}(T)=(n+1)\psi_{n+1}(T)-n\psi_n(T) \quad (31)$$

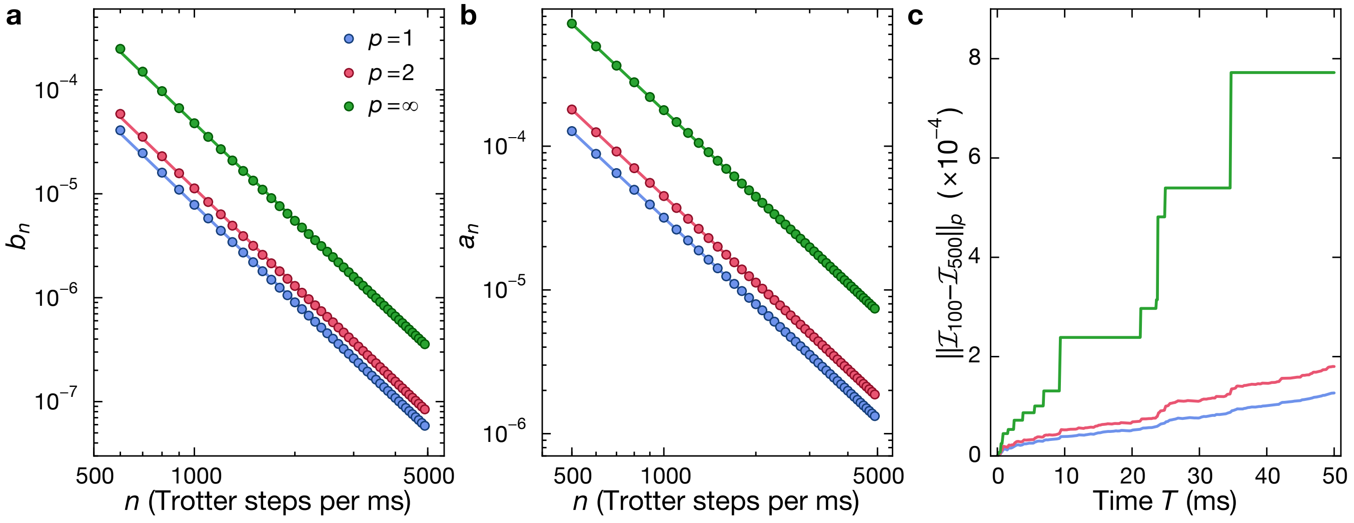

Supplementary Figure 13. **Error estimates in Supplementary Eq.** (29)**.** In panels **a** and **b**, the $p-$norms are calculated for $T = 100\,\text{ms}$ **a** Computation of $b_n = ||\mathcal{I}_n - \mathcal{I}_{n+r}||_p$ with $r = 100$ for $L = 8, N_\sigma = 2$. We show b_n for $p = 1, 2$ and ∞. The solid lines are power law fits, and the corresponding exponent is about -3.09. **b** Computation of $a_n = ||\mathcal{I} - \mathcal{I}_n||_p$ for a system with $L = 8, N_\sigma = 2$. The solid lines correspond to the respective estimates of $\mathcal{I}(t)$ given by Supplementary Eq. (30). The three colors represent $p = 1, 2$ and ∞. **c** The growth of the errors $||\mathcal{I}_{100} - \mathcal{I}_{500}||_p$ in time for $p = 1, 2$ and ∞, for $L = 8$ and $N_\sigma = 2$.

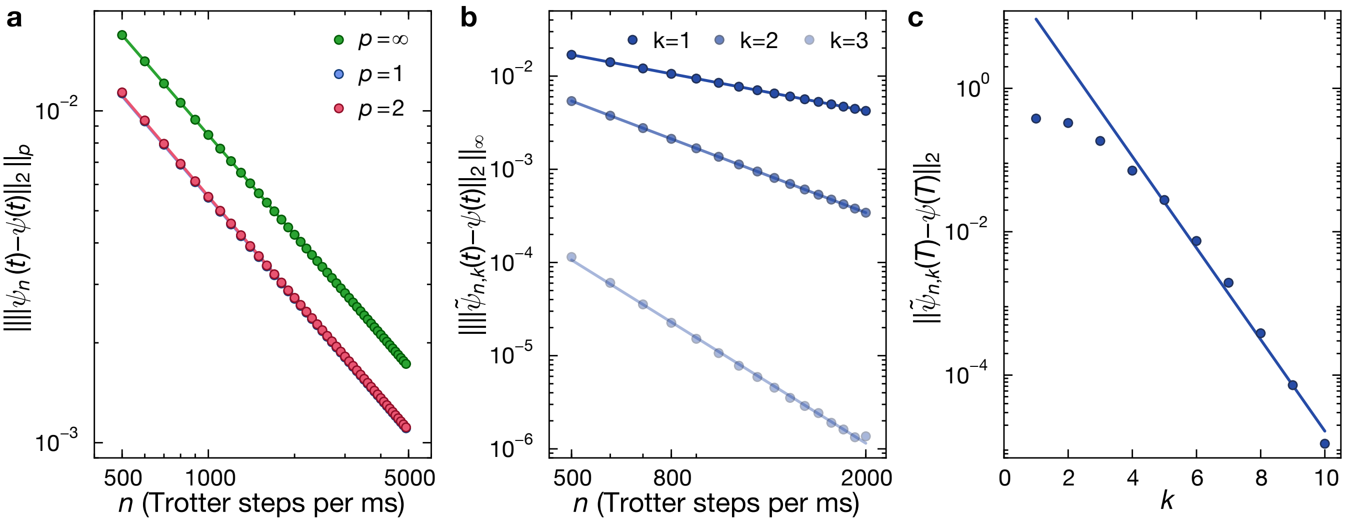

Supplementary Figure 14. **General error analysis.** **a** $||||\psi_n(t) - \psi(t)||_2||_p$ for a system with $L = 8, N_\sigma = 2$, indicating a convergence rate of $\frac{1}{n}$. **b** $||||\tilde{\psi}_{n,k}(t) - \psi(t)||_2||_\infty$ for different $k = 1, 2, 3$, system size $L = 8$ and $N_\sigma = 2$ with $T = 100\,\text{ms}$. $k = 2$ corresponds to the elimination of the first order error following Supplementary Eq. (31). The $k = 3$ curve corresponds to an elimination of the first two orders in error. The straight lines are power law fits with exponents -0.99, -1.99 and -3.26 respectively for $k = 1, 2$ and 3, indicating a convergence rate of $\frac{1}{n^k}$. **c** $||\tilde{\psi}_{n,k}(T) - \psi(T)||_2$ at $T = 100\,\text{ms}$ for a system with $L = 8, N_\sigma = 2$ and $n_1 = 40$ for various k, indicating a convergence rate of $\frac{1}{n_1^k}$.

$\tilde{\psi}_{n,2}$ is an attempt to eliminate the first order term in the error and therefore, we expect the error scaling to be lower for this state. Indeed, as shown in Supplementary Fig. 14b, $||\tilde{\psi}_{n,2} - \psi(T)|| \sim 1/n^2$. We may consider a general procedure to eliminate the higher order error terms. We pick k integers $n_1, \cdots, n_k$ and compute $\psi_{n_1}(T), \cdots, \psi_{n_k}(T)$ independently and eliminate the first $k-1$ orders of error. This is done using the Vandermonde matrix

$$W_k = \begin{pmatrix} 1 & 1/n_1 & \cdots & 1/n_1^{k-1} \\ 1 & 1/n_2 & \cdots & 1/n_2^{k-1} \\ \vdots & \vdots & \ddots & \vdots \\ 1 & 1/n_k & \cdots & 1/n_k^{k-1} \end{pmatrix}$$

We can eliminate the errors using the expression $\tilde{\psi}_{n_1,k} = \sum_j (W_k^{-1})_{1j} \psi_{n_j}(T)$. Supplementary Fig. 14c

shows that the error in $\tilde{\psi}_{n_1,k}$ scales down as $\sim 1/n_1^k$.

[1] C. Parra-Murillo and S. Wimberger, Manifold Approach for a Many-Body Wannier-Stark System: Localization and Chaos in Energy Space, Acta Physica Polonica A **124** (2014).

[2] C. A. Parra-Murillo, J. Madroñero, and S. Wimberger, Two-band Bose-Hubbard model for many-body resonant tunneling in the Wannier-Stark system, Phys. Rev. A **88**, 032119 (2013).

[3] D. V. Else, W. W. Ho, and P. T. Dumitrescu, Long-Lived Interacting Phases of Matter Protected by Multiple Time-Translation Symmetries in Quasiperiodically Driven Systems, Phys. Rev. X **10**, 021032 (2020).

[4] E. v. Nieuwenburg, Y. Baum, and G. Refael, From Bloch oscillations to many-body localization in clean interacting systems, PNAS **116**, 9269 (2019).

[5] N. Goldman and J. Dalibard, Periodically Driven Quantum Systems: Effective Hamiltonians and Engineered Gauge Fields, Phys. Rev. X **4**, 031027 (2014).

[6] A. Eckardt and E. Anisimovas, High-frequency approximation for periodically driven quantum systems from a Floquet-space perspective, New Journal of Physics **17**, 093039 (2015).

[7] M. Bukov, L. D'Alessio, and A. Polkovnikov, Universal high-frequency behavior of periodically driven systems: from dynamical stabilization to Floquet engineering, Adv. Phys. **64**, 139 (2015).

[8] D. A. Abanin, W. De Roeck, and F. Huveneers, Exponentially Slow Heating in Periodically Driven Many-Body Systems, Phys. Rev. Lett. **115**, 256803 (2015).

[9] L. D'Alessio and M. Rigol, Long-time Behavior of Isolated Periodically Driven Interacting Lattice Systems, Physical Review X **4** (2014).

[10] D. A. Abanin, W. De Roeck, W. W. Ho, and F. Huveneers, Effective Hamiltonians, prethermalization, and slow energy absorption in periodically driven many-body systems, Phys. Rev. B **95**, 014112 (2017).

[11] W. De Roeck and V. Verreet, Very slow heating for weakly driven quantum many-body systems, arXiv:1911.01998 (2019).

[12] D. V. Else, B. Bauer, and C. Nayak, Prethermal Phases of Matter Protected by Time-Translation Symmetry, Phys. Rev. X **7**, 011026 (2017).

[13] T. Kuwahara, T. Mori, and K. Saito, Floquet–Magnus theory and generic transient dynamics in periodically driven many-body quantum systems, Annals of Physics **367**, 96–124 (2016).

[14] This discussion would also apply in the limit of incommensurate Δ_σ and U when going to the frame with respect to the interacting term.

[15] U. Schneider, L. Hackermüller, J. P. Ronzheimer, S. Will, S. Braun, T. Best, I. Bloch, E. Demler, S. Mandt, D. Rasch, and A. Rosch, Fermionic transport and out-of-equilibrium dynamics in a homogeneous Hubbard model with ultracold atoms, Nature Physics **8**, 213–218 (2012).

[16] S. Bravyi, D. P. DiVincenzo, and D. Loss, Schrieffer–Wolff transformation for quantum many-body systems, Annals of Physics **326**, 2793–2826 (2011).

[17] E. S. Mananga and T. Charpentier, Introduction of the Floquet-Magnus expansion in solid-state nuclear magnetic resonance spectroscopy, The Journal of Chemical Physics **135**, 044109 (2011).

[18] T. Mikami, S. Kitamura, K. Yasuda, N. Tsuji, T. Oka, and H. Aoki, Brillouin-Wigner theory for high-frequency expansion in periodically driven systems: Application to Floquet topological insulators, Phys. Rev. B **93**, 144307 (2016).

[19] P. Sala, T. Rakovszky, R. Verresen, M. Knap, and F. Pollmann, Ergodicity Breaking Arising from Hilbert Space Fragmentation in Dipole-Conserving Hamiltonians, Phys. Rev. X **10**, 011047 (2020).

[20] V. Khemani, M. Hermele, and R. Nandkishore, Localization from Hilbert space shattering: From theory to physical realizations, Phys. Rev. B **101**, 174204 (2020).

[21] S. R. Taylor, M. Schulz, F. Pollmann, and R. Moessner, Experimental probes of Stark many-body localization, Phys. Rev. B **102**, 054206 (2020).

[22] D. Abanin, W. De Roeck, W. W. Ho, and F. Huveneers, A Rigorous Theory of Many-Body Prethermalization for Periodically Driven and Closed Quantum Systems, Commun. Math. Phys. **354**, 809 (2017).

[23] R. Sensarma, D. Pekker, E. Altman, E. Demler, N. Strohmaier, D. Greif, R. Jördens, L. Tarruell, H. Moritz, and T. Esslinger, Lifetime of double occupancies in the Fermi-Hubbard model, Phys. Rev. B **82**, 224302 (2010).

[24] N. M. R. Peres, R. G. Dias, P. D. Sacramento, and J. M. P. Carmelo, Finite-temperature transport in finite-size Hubbard rings in the strong-coupling limit, Phys. Rev. B **61**, 5169 (2000).

[25] C.-J. Lin and O. I. Motrunich, Explicit construction of quasiconserved local operator of translationally invariant nonintegrable quantum spin chain in prethermalization, Phys. Rev. B **96**, 214301 (2017).

[26] S. Moudgalya, A. Prem, R. Nandkishore, N. Regnault, and B. A. Bernevig, Thermalization and its absence within Krylov subspaces of a constrained Hamiltonian, arXiv:1910.14048 (2019).

[27] T. Rakovszky, P. Sala, R. Verresen, M. Knap, and F. Pollmann, Statistical localization: From strong fragmentation to strong edge modes, Phys. Rev. B **101**, 125126 (2020).

[28] C. E. Soliverez, An effective Hamiltonian and time-independent perturbation theory, Journal of Physics C: Solid State Physics **2**, 2161 (1969).

[29] T. Kato, On the Convergence of the Perturbation Method. I, Progress of Theoretical Physics **4**, 514 (1949).

[30] D. Prato and P. W. Lamberti, A note on Magnus formula, The Journal of Chemical Physics **106**, 4640 (1997).

[31] D. N. Page, Average entropy of a subsystem, Phys. Rev. Lett. **71**, 1291 (1993).

[32] L. Vidmar and M. Rigol, Entanglement Entropy of Eigenstates of Quantum Chaotic Hamiltonians, Phys. Rev. Lett. **119** (2017).

[33] G. De Tomasi and I. M. Khaymovich, Multifractality meets entanglement: Relation for nonergodic extended states, Phys. Rev. Lett. **124** (2020).

[34] M. Schreiber, S. S. Hodgman, P. Bordia, H. P. Lüschen, M. H. Fischer, R. Vosk, E. Altman, U. Schneider, and

I. Bloch, Observation of many-body localization of interacting fermions in a quasirandom optical lattice, Science **349**, 842 (2015).

[35] S. Scherg, T. Kohlert, J. Herbrych, J. Stolpp, P. Bordia, U. Schneider, F. Heidrich-Meisner, I. Bloch, and M. Aidelsburger, Nonequilibrium Mass Transport in the 1D Fermi-Hubbard Model, Phys. Rev. Lett. **121**, 130402 (2018).

[36] J. Sebby-Strabley, M. Anderlini, P. S. Jessen, and J. V. Porto, Lattice of double wells for manipulating pairs of cold atoms, Phys. Rev. A **73**, 033605 (2006).

[37] S. Fölling, S. Trotzky, P. Cheinet, M. Feld, R. Saers, A. Widera, T. Müller, and I. Bloch, Direct observation of second-order atom tunnelling, Nature **448** (2007).

[38] C. A. Regal, M. Greiner, and D. S. Jin, Observation of Resonance Condensation of Fermionic Atom Pairs, Phys. Rev. Lett. **92**, 040403 (2004).

[39] C. A. Regal and D. S. Jin, Measurement of Positive and Negative Scattering Lengths in a Fermi Gas of Atoms, Phys. Rev. Lett. **90**, 230404 (2003).

[40] R. Jördens, *Metallic and Mott-Insulating Phases in Fermionic Quantum Gases*, Ph.D. thesis, ETH Zurich, Zürich (2010).
